# Emergence of Cardiac Glycosides as Potential Drugs: Current and Future Scope for Cancer Therapeutics

**DOI:** 10.3390/biom11091275

**Published:** 2021-08-25

**Authors:** Ranjith Kumavath, Sayan Paul, Honey Pavithran, Manash K. Paul, Preetam Ghosh, Debmalya Barh, Vasco Azevedo

**Affiliations:** 1 Department of Genomic Science, School of Biological Sciences, Central University of Kerala, Tejaswini Hills, Periya (P.O) Kasaragod, Kerala 671320, India; honeypavithran6@gmail.com; 2 Department of Biotechnology, Manonmaniam Sundaranar University, Tirunelveli, Tamilnadu 627012, India; sayanpaul01@yahoo.com; 3 Centre for Cardiovascular Biology and Disease, Institute for Stem Cell Science and Regenerative Medicine, Bangalore 560065, India; 4 Department of Pulmonary and Critical Care Medicine, David Geffen School of Medicine, UCLA, Los Angeles, CA 90095, USA; paul_cancerbiotech@yahoo.co.in; 5 Department of Computer Science, Virginia Commonwealth University, Richmond, VA 23284, USA; preetam.ghosh@gmail.com; 6 Institute of Integrative Omics and Applied Biotechnology (IIOAB), Nonakuri, Purba Medinipur 721172, India; dr.barh@gmail.com; 7 Laboratório de Genética Celular e Molecular, Departamento de Genetica, Ecologia e Evolucao, Instituto de Ciências Biológicas, Universidade Federal de Minas Gerais, Belo Horizonte, Minas Gerais 31270-001, Brazil; vascoariston@gmail.com

**Keywords:** cardiac glycosides, transcription factors, therapeutic target, cancer therapy, in vitro and in vivo anticancer activities

## Abstract

Cardiac glycosides are natural sterols and constitute a group of secondary metabolites isolated from plants and animals. These cardiotonic agents are well recognized and accepted in the treatment of various cardiac diseases as they can increase the rate of cardiac contractions by acting on the cellular sodium potassium ATPase pump. However, a growing number of recent efforts were focused on exploring the antitumor and antiviral potential of these compounds. Several reports suggest their antitumor properties and hence, today cardiac glycosides (CG) represent the most diversified naturally derived compounds strongly recommended for the treatment of various cancers. Mutated or dysregulated transcription factors have also gained prominence as potential therapeutic targets that can be selectively targeted. Thus, we have explored the recent advances in CGs mediated cancer scope and have considered various signaling pathways, molecular aberration, transcription factors (TFs), and oncogenic genes to highlight potential therapeutic targets in cancer management.

## 1. Introduction

Globally, the incidence and mortality of cancer are forecast to rise to 19.3 million cases and 10 million deaths in 2021, making it the second most prevalent cause of death. According to recent reports, female breast cancer (11.7%) has been the most commonly diagnosed cancer, followed by lung (11.4%), colorectal (10%), prostate (7.3%), and stomach (5.6%) cancers. Lung cancer is the highest cause of patient deaths, with an estimated 1.8 million deaths (18%), followed by colorectal (9.4), liver (8.3%), stomach (7.7%), and breast (6.9%) cancers [1]. Some common reasons for cancer include spontaneous or environmentally-induced accumulation of genetic and epigenetic aberrations in the genome, leading to an uncontrolled proliferation of tumor cells [2,3]. It can also result from dysregulation in transcription regulating network proteins such as transcription factors (TFs). TFs are among the most prominent regulatory proteins that bind to specific DNA sequences and regulate gene expression by influencing RNA polymerase activity [4,5]. Thus, researchers worldwide are exploring novel therapeutics that can target these unique mutations in the genome while avoiding the systemic toxicity typical of the traditional chemotherapeutic approach.

In the search for novel therapeutic agents for cancer treatment, natural compounds have always been a focus of scientific attention, and this interest continues to grow. Interest in natural compounds is attributed to several factors such as the minimal side effects compared to chemo or radiation therapy, an extensive array of chemical diversity, and the ease of access and bioprocessing [6,7]. Natural bioactive fractions and pure compounds derived from plants are widely used to treat multiple cancers [8]. In recent years, myriads of plant-derived compounds with anticancer properties were identified that could specifically act on various pathways to inhibit carcinogenesis. Some phytochemically derived anticancer compounds like calebin A, nobiletin, garcinol, CG, and many others target TFs precisely [9,10,11,12]. Cardiac glycosides (CGs) are of particular interest because of their potential as drug repurposing candidates derived from plant and animal sources and are used in various human ailments [13]. CGs are principally involved in the inhibition of the Na^+^/K^+^ ATPase pump during muscle contraction in heart failure, and thus, they are clinically approved for the treatment of cardiovascular diseases [14]. Interestingly, the roles of CGs have also been explored for broad application in various cancers. CGs exhibit selective cytotoxic effects on tumor cells; thus, their potential for use as anticancer molecules has increased [15]. Some of the potent inhibitors of cancer cell growth from the group include digitoxin, peruvoside, strophanthidin, bufalin, and ouabain; the principal use of CGs today comes from their ability to inhibit Na^+^/K^+^ ATPase pump. However, studies related to the specificity of Na^+^/K^+^ ATPase pump in cancer cells have primarily been undertaken to identify its impact on immunogenic cell death that stimulates the cognate immune response.

Furthermore, recent studies have delineated that CGs exhibit an immense potential to directly or indirectly inhibit the activation of the TFs such as NF-κB, FGF-2, STAT-3, HIF-1α, and thus, suppress tumor growth [16,17]. This review pursues the recent insights into the therapeutic strategies of targeting signaling pathways in cancer and emphasizes the importance of natural agents that can regulate the aberrations caused. We discuss below the functional and therapeutic scope of CGs and their role in modulating various cancer signaling pathways and dysregulations in TF activity, thus providing new insights into their anticancer activities. We also identify the functional potential of CGs in inhibiting tumor growth.

## 2. Cardiac Glycosides

CGs are widely distributed naturally-derived cardiotonic steroids obtained predominantly from various plants and amphibian sources and considered highly useful in cardiac and cancer therapeutics [18,19]. Structurally the CG drugs are composed of glycone (sugar) and aglycone (steroid) moieties [20]. The core structure of the CG is characterized by the presence of lactone-containing substituent at the β-17 position (butyrolactone or α-pyrone) and a sugar moiety at the β-3 position of the steroidal core [21]. The CGs aglycone moiety is composed of a steroidal nucleus, and the lactone ring of the aglycone moiety regulates the functional activity of the CG [22]. Besides, the glycone moiety regulates the toxicokinetic and toxicodynamic profiles of the CGs. The most commonly occurring sugars within the glycone moieties are glucose, fructose, galactose, glucuronide, mannose, rhamnose, and digitalose [23,24,25]. The overall potency of the CGs is influenced by the type of sugar attached to the steroid [23]. The addition of the rhamnose was reported to enhance the potency of the CG compound 6 to 35 times. In contrast, the addition of mannose did not exhibit any significant effect on potency [26]. Depending on the R group at position 17, the CGs are structurally classified into two types: (i) cardenolides and (ii) bufadienolides [20]. The cardenolides (lactone 2-furanone) are characterized by the presence of unsaturated five-membered butyrolactone ring at position C-17, and the bufadienolides (lactone 2-pyrone) are identified by the presence of the doubly unsaturated six-member α-pyrone ring.

Previous literature has documented the presence of CGs in the seed, leaf, stem, root, and bark of several plant species, acting as a significant source of arrow poisons and widely distributed in Africa, Asia, and South America. The cardenolides were confined to the angiosperms and abundantly present in Apocynaceae and Asclepiadaceae [20]. Additionally, they were also reported to occur in Brassicaceae, Fabaceae, Malvaceae, Solanaceae, Cruciferae, Sterculiaceae, and Euphorbiaceae [27]. Furthermore, the bufadienolides were found in the plant families, including Crassulaceae, Iridaceae, Hyacinthaceae, Melianthaceae, Ranunculaceae, Santalaceae, and animal sources including toads, fireflies, and snakes [27]. Some of the widely known and characterized CGs include digoxin, digitoxin, ouabain, oleandrin, calotropin, thevetin, convallatoxin, bufalin, marinobufagenin, and telocinobufagin [20,25]. The cardenolides like digoxin and digitoxin were derived from the foxglove species *Digitalis lanata* and *Digitalis purpurea*. The African arrow poison ouabain was found in species belonging to the genera Acokanthera and Strophanthus. The toxic oleandrin was obtained from the Apocynaceae plant species called *Nerium oleander* [28]. The milkweed species belong to Asclepias, and the family Asclepiadaceae, which is reported to produce the toxic cardenolide calotropin. Besides, a poisonous CG like thevetin was found in the South American ornamental shrub *Cascabela thevetioides* (Kunth) [29], and convallatoxin was reported in the plant *Convallaria majalis* [30]. The cardiotonic steroid bufalin was found in the dried venom obtained from the parotoid gland of the Chinese bufo toad, and steroids including marinobufagenin and telocinobufagin were isolated from the skin sample secretions of the Brazilian toad, *Bufo rubescens*, and the giant neotropical marine toad *Rhinella marina* [31,32].

## 3. Mode of Action of Cardiac Glycosides

**Figure 1:**
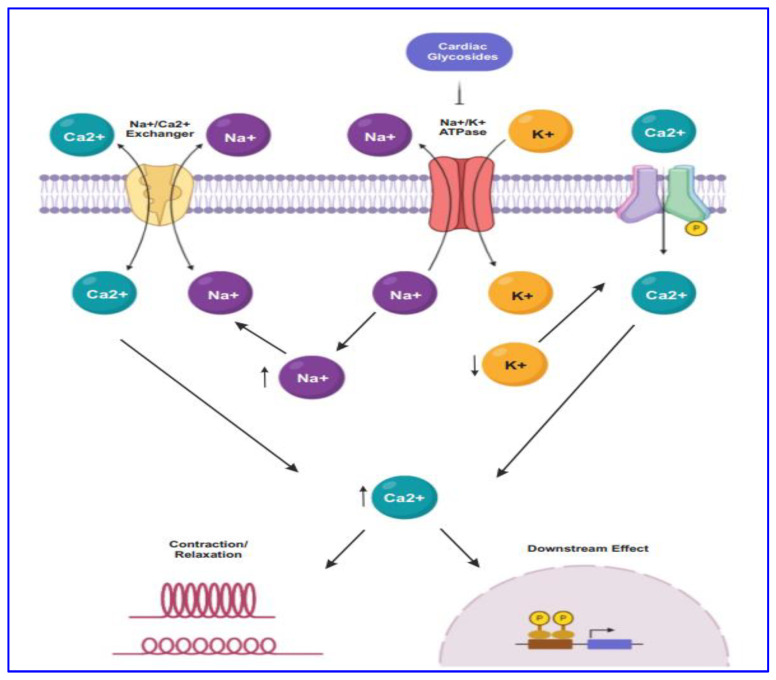
The mode of action of CGs in cancer proceeds through targeting Na^+^/K^+^-ATPase by maintaining the concentration of sodium-potassium gradient across the plasma membrane. CG binds to the Na^+^/K^+^-ATPase pump, thus inhibiting it, resulting in intracellular retention of Na^+^ and increasing the concentration of Ca^2+^. Subsequently, lower expression of Na^+^/K^+^-ATPase causes endoplasmic reticulum stress.

For several decades CG drugs were used in cardiology as folk medicines, diuretics, and emetics to treat cardiac congestion and cardiac arrhythmia [25]. The CG steroids affect cardiac contractility by targeting the cellular Na^+^/K^+^ ATPase pump. The inhibition of Na^+^/K^+^ ATPase pump (Figure 1) leads to intracellular retention of Na^+^ and subsequently induces the concentration of intracellular Ca^2+^ ion mediated by the effect of Na^+^/Ca^2+^ membrane exchanges [33]. The elevated level of intracellular Ca^2+^ concentration causes inotropy and bradycardia. Besides, the accumulation of intracellular Na^+^ and Ca^2+^ result in the membrane and ventricular ectopy [33].

## 4. Modulation of Transcription Factor Activity through Cardiac Glycosides

There has been resurgence in the study of transcription and its regulators in cancer during the last several years. Reports suggest that aberrant gene expression is a fundamental prerequisite for cancer cells to maintain their enhanced metabolism and proliferative state [34,35,36]. Hence these dysregulated cellular processes involving several druggable proteins can be targeted through various disrupters for cancer drug development. Thus, here we have gathered the study reports, illustrating the effect of CGs in controlling these processes through their regulators that make them excellent metabolic targets in cancer therapy.

Mutated transcription factors represent a unique class of drug targets. They are the crucial regulators that regulate the transcriptional process and thus control the rate of transcription of the genetic information [37]. In recent years the scientists has identified the scope of targeting these factors and thus regulating cancer growth by blocking the transcription of undesired or oncogenic genes. Some of the key TFs that act as drivers of cancers include NF-kB, HIF1, C-Myc, AP-1, STAT3, etc. [38].

Several reports describe the increased activity of transcription factor NF-kB in various cancers, including breast, lung, colon, and thyroid cancers [39,40,41]. Giuliani and their group [42] illustrated the role of (NF-kB) and its regulation of other genes involved in cell growth, survival, proliferation, and differentiation. Furthermore, they also showed the significance of mutated oncogenes and tumor suppressive genes in NF-kB activation, thus causing aggressive patterns of the disease. Hypoxia-inducible factor 1 (HIF1) is a predominant TF that activates the transcription of downstream genes that mediates cancer-promoting consequences such as increased cell proliferation, survival, invasion, and metastasis [43]. The HIF-1α unit of the protein is recurrently overexpressed in many human cancers due to genetic alteration or mutation of oncogenes. Furthermore, pathway delineation has provided supporting evidence stating that these HIF1 are in turn regulated by PI3K and MAPK pathway proteins [44] which again plays a significant role in cancer. Following the initial findings, a comprehensive screening of drugs as inhibitors of (HIF1) through clinical trials found that CGs, including digitoxin could inhibit HIF-1 and downstream genes in the process of transcription [45].

**Figure 2:**
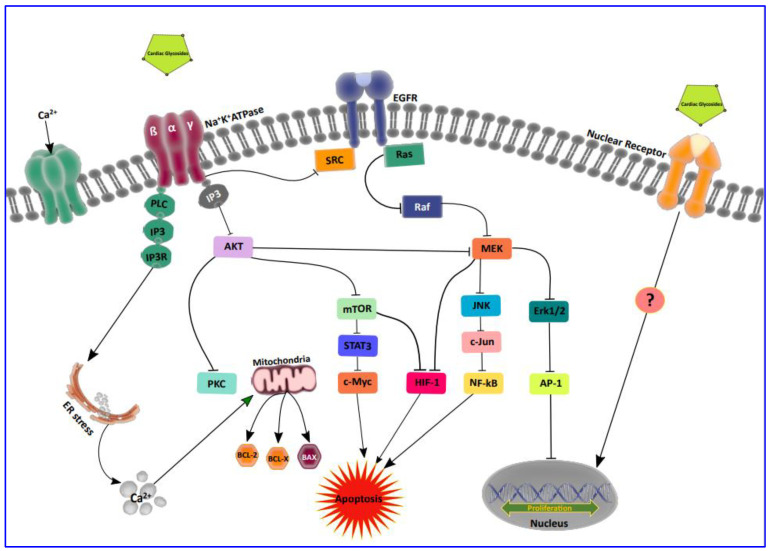
Illustrating the inhibition of transcription factors activated in cancer through cardiac glycosides. The binding of CGs on the Na^+^/K^+^/ATPase (NKA) channel decreases the NKA pump activity and thus down regulates multiple signal transduction cascades especially targeting the TF proteins involved in the cell growth and cell proliferation by providing signals to the transcription machinery. Dysregulated TF and their targeted therapy through CG are represented in the pathway. The scope of targeting nuclear receptors via CG is an upcoming purview to be studied for therapeutic potential.

The predominant role of activating protein (AP-1) in self-sufficient proliferation and migration was recently reported through an intensive study process carried out by Ibrahim [46] and his group. They highlighted the effect of Fos and c-Jun proteins, the members of the AP-1 family, and its prevalence in the human breast and lung cancer signaling cascade [46]. Exciting findings of Prassas and Diamandis [47] showed that the mode of action of CG on Na^+^/K^+^-ATPase can alter the function of AP-1 and thus regulate transcriptional gene processes. Furthermore, through a transient increase in the intracellular Ca^2+^, CGs can modulate the NF-kB and protein kinase (PKC) activity which further affects AP-1, thus causing a decrease in cell proliferation and increasing apoptosis in tumor cells [48,49]. There are also reports that AP-1 regulates cyclin-D1 and C-Myc transcription in an AKT-dependent manner in response to mTOR inhibition through CGs. Moreover, it is a conventional fact that CG exerts antitumor activity profoundly through PI3k/AKT/mTOR signal transduction inhibition [50]. Recently, it has been shown that CGs impart direct and indirect interactions with nuclear receptors, which in turn are involved in the transcription of growth-related genes. These TFs interact with other proteins involved in the general transcription machinery such as TBP (TATA-binding protein), coactivators and corepressors, etc. [13]. However, the exact role played by CGs on these nuclear receptors is yet to be proven. Thus, compiling all of this information, we present our current understanding of the targeted therapeutic potential of transcription factors and the role of CGs in modulating these proteins in cancer as depicted in Figure 2.

## 5. Anti-Proliferative and Cytotoxic Effects of Cardiac Glycosides

**Figure 3:**
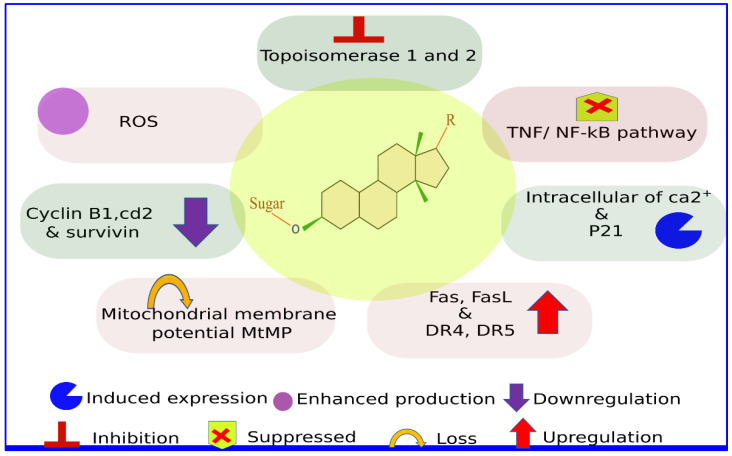
Molecular mechanisms for the antiproliferative and cytotoxic effects of cardiac glycosides.

Uncontrolled cell proliferation is considered as a significant hallmark of cancer cells [51]. Cancer progression is characterized by continuing cell proliferation leading to tumor development and rapid expansion [51,52]. Unlike normal cells, cancer cells do not rely on external stimulators like growth factors for their growth and division [51]. The cancer cells can also evade the growth suppressors and antiproliferative signals, which drive inadequate cell division and dysregulate tissue homeostasis. Several studies have highlighted the antiproliferative activity of CG drugs on different cancer cells (Figure 3); bufalin showed antiproliferation against human melanoma BRO cells by arresting them at the G2/M phase. Jiang et al. have reported that bufalin exerts its antiproliferative effect on A459 non-small cell lung cancer (NSCLC) cells in a time-dependent manner [53], where bufalin reduced the cellular viability of A549 cells by enhancing the expression of p53 and p21 (WAF1/CIP1) genes. Besides, bufalin inhibits the expression of Cyclin D1 by 50% in the A549 cells. The tumor suppressor gene p21 forms a heterotrimeric complex with cyclin and cyclin-dependent kinase to reduce the kinase activity and arresting the cell cycle progression through the G1/S phase [53].

The antineoplastic effects of digitoxin and synthetic analog D6-MA on the NSCLC and NCI-H460 cell lines have been reported previously. Digitoxin and D6-MA arrest the cell cycle at the G2/M phase by down regulating the expression of molecular drivers of the G2/M phase, including cyclin B1, cdc2, and survivin [54]. Cyclin B1 and cdc2 form cyclin B1/cdc2 complex, which regulates the progression of the cell cycle through the G2/M phase and protects the mitotic cells from apoptosis [55,56,57]. Besides, survivin acts as a key regulator of mitosis by activating the chromosomal passenger complex [58]. The digitoxin and D6-MA exposure lead to the down regulation of p53, p21, p27, and checkpoint kinases Chk1 and Chk2 in a dose-dependent manner [54]. The p53 protein acts as a critical coordinator of cell cycle checkpoints for effective apoptosis [59]. p21 protein promotes mitosis and cell proliferation and is associated with several cancer conditions; nonetheless, the down regulation of p21 by digitoxin is essential to control the cell cycle progression and reduce cell viability. Recently Silva et al. [60] demonstrated that amantadig, a semisynthetic cardenolide derivative of digitoxigenin, in combination with docetaxel exhibited a synergistic anti-proliferative effect on human androgen-insensitive prostate cancer cells DU145 and PC-3.

The antiproliferative effect of ouabain in human breast (BT20) and prostate (DU145) cancer cell lines [61]; ouabain treatment led to degradation of Na/K-ATPase mediated by endocytosis and induced the expression of cell cycle inhibitor p21Clp1, which inhibited the proliferation of the cells. The inhibitory effect of digitoxin, digoxin, and ouabain was reported on the androgen-dependent LNCaP, androgen-independent DU145, and PC3 cells in a dose and time-dependent manner [62]. Of these three CGs, ouabain more effectively exerted its antiproliferative effect on prostate cancer cells than digoxin and digitoxin. Besides, the androgen-dependent LNCaP cells were more sensitive to digitalis treatment than other cell lines and induced intracellular Ca^2+^ influx by two to four-fold, leading to cell toxicity, and apoptosis by activating several hydrolytic enzymes, proteases, nucleases, and lipases [62]. The CGs differ significantly in terms of their cytotoxicity, potency, and selectivity. Johansson et al. investigated the cytotoxic effect of five CG on the primary cultures of human tumor cells [63]. The data suggested that proscillaridin A was the most potent and cytotoxic compound, followed by digitoxin, ouabain, digoxin, and lanatoside C. Both digitoxin and digoxin exhibited selective cytotoxicity against the solid tumor cells. In contrast, proscillaridin A lacked selective cytotoxicity towards solid and hematological tumor cells.

Oleandrin acts as a potential suppressor of TNF-induced NF-kB activation in a concentration and time-dependent manner [48]. The cardiotonic drug blocked the activation of TNF-induced AP-1 (activator protein 1), JNK, and MEK. The TNF-induced activation of NF-kB requires the TNF receptor’s sequential interaction with TRADD, TRAF2, NIK, and IKK-β. The IKK-β acts as an inhibitor of NF-kB [48]. The oleandrin treatment significantly blocked the activity of TRAF2 and NIK and played a crucial role in antagonizing both phosphorylation and degradation of IKK-β.

## 6. Role of Cardiac Glycosides in Apoptosis

Several studies have documented that caspases, a cysteinyl aspartate specific protease family, play a key role in cellular apoptosis. Apoptosis is primarily mediated by pathways, namely mitochondria-mediated and death-receptor-mediated. The activation of caspase cascades significantly differs between both pathways. The extrinsic pathway led to the induced level of DISC formation in the activation of caspase-8 [64]. Activated caspase-8, in turn, triggers downstream effector caspase-3/7. The intrinsic pathway results in the release of cytochrome c from the mitochondria, which causes apoptosome formation and activation of procaspase-9 [64,65]. The activated caspase-9, in turn, activates its downstream effector caspase-3/7. The apoptotic activity of oleanen regulates the human cervical cancer HeLa cells through the intrinsic apoptosis pathway. The oleanen triggered caspase 3/7, caspase 6, and caspase 9, respectively, in a dose dependent manner. Besides, it triggers endogenous apoptotic pathway by up regulating pro-apoptotic factor BIM [66].

Elbaz et al. [54] discussed the role of digitoxin in regulating cellular apoptosis in human NSCLC (NCI-H460) cells. The digitoxin treatment induced the apoptosis of NCI-H460 cells by triggering the cleavage of caspase-9, mediated through the intrinsic mitochondrial pathway. Besides, the digitoxin treatment led to survivins down regulation, an inhibitor of caspase-9 activity. Previous studies have demonstrated that the Fas/Fas ligand (FasL) and TNF-related apoptosis-inducing ligand are crucial mediators of apoptosis through the extrinsic pathway mechanism [67]. Deregulation of these ligand molecules resulted in the immune escape against several cancers and resistance to anticancer drugs [68]. Sreenivasan et al. [69] noted that the cardiac glycoside oleandrin upregulated the Fas expression and attenuated the NF-kB activation in the tumor cells without affecting the primary cells. NF-kB is also reported as a key pathway that exerts anti-apoptotic and pro-proliferative effects [70]. Hence, the NF-kB attenuation played a key role in triggering apoptosis in the tumor cell lines. The CGs such as oleandrin, digitoxin, bufalin, and digoxin sensitized lung cancer cells to Apo2L/TRAIL-induced apoptosis by upregulating the expression of death receptors 4 and 5 at both RNA and protein levels [71]. Chanvorachote et al. [72] reported that the CG drug ouabain sensitizes the lung cancer cells H292 to TRAIL-induced apoptosis by down-regulating the expression of Mcl-1. The Mcl-1 gene plays a crucial role in repressing the TRAIL-mediated apoptosis. Hence, it is evident that the down regulation of the gene modulates the viability of lung cancer cells.

**Figure 4:**
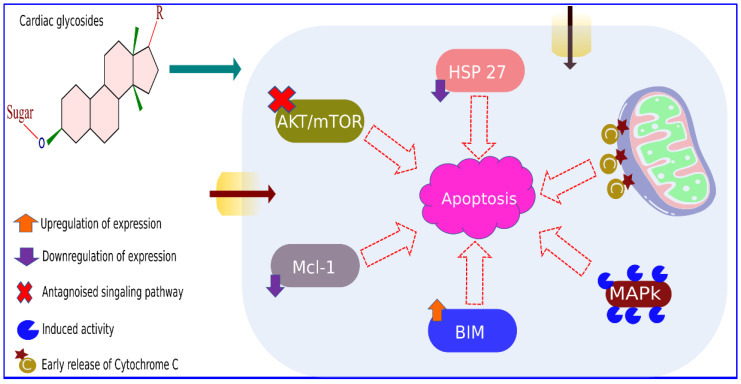
Molecular mechanisms associated with cardiac glycoside induced apoptosis.

The cardiotonic steroid ouabain showed cytotoxic activity and induced apoptosis against the human neuroblastoma (SH-SY5Y) cell line. The ouabain exposure stimulated the concentration-dependent phosphorylation of Erk1/2, Akt, and Bad. Besides, ouabain downregulated the expression of anti-apoptotic proteins Bcl-XL and Bcl-2, resulting in the release of cytochrome c into cytosol and induced caspase expressions to trigger cellular apoptosis in SH-SY5Y cells [73]. The UNBS1450 treatment at low nanomolar concentrations triggered apoptotic cell death by inhibiting NF-kB transactivation and inducing the cleavage of pro-caspases 8, 9, and 3/7. Besides, UNBS1450 treatment downregulated the expression of the anti-apoptotic protein (Mcl-1), and up-regulated the level of pro-apoptotic proteins Bak and Bax, which eventually led to cell death [74]. The molecular mechanism associated with CG-induced apoptosis is documented in Figure 4 and Table 1. A recent study by Geng et al. [75] demonstrated that cardiac glycoside inhibits cancer cell progression by inducing the Na^+^/K^+^ ATPase-dependent cell death.

## 7. Cardiac Glycosides as Cancer Therapeutics

Over the years, several reports have documented the cancer therapeutic potential of CGs (Table 2). The first epidemiological evidence regarding the anticancer effect of CGs was provided by Stenkvist et al. [129]. A series of studies confirmed that the breast cancer tissue samples obtained from the patients treated with digitalis CG therapy exhibited more delicate features than the cancer samples from control patients without digitalis therapy. The risk of cancer recurrence post five years of mastectomy was 9.5 times higher in the control patients without digitalis therapy than the patients treated with digitalis. The study indicated the significant impact of CGs on the biological aggressiveness of breast cancer. Subsequently, Goldin and Safa, in 1984 [130], screened the effect of digitalis on the mortality rate of 127 breast cancer patients. The study documented the death of 21 patients due to cancer, and among them, only one patient was previously treated with digitalis. The report confirmed the potential rate of digitalis in protecting against cancer.

In a long-term follow-up of 22.3 years Stenkvist [136] reported that digitalis plays a therapeutic role in reducing mortality in breast cancer patients. In his study spanning 175 breast carcinoma patients, 32 patients received digitalis therapy. Of them, only two patients died due to cancer (6%). In contrast, the control group containing 143 patients without digitalis therapy showed a mortality rate of 34%. Haux et al. [137] performed internal dose-response analysis using the plasma of the 9271 patients treated with digitoxin and investigated the CG compound’s antineoplastic effect on leukemia and kidney/urinary tract cancer. Comparison between high plasma concentration of digitoxin and reduced risk for lymph proliferative and urinary tract cancers yielded encouraging in vitro and in vivo results, establishing the role of CGs as crucial anticancer compounds.

### 7.1. Cardiac Glycosides Effects on Anticancer Properties by In Vitro Studies

The first in vitro evidence regarding the cytotoxic effects of CG on human neoplastic Hela-S3 cells was reported in 1967 by Shiratori [138]. Subsequently, numerous studies were conducted to demonstrate the antiproliferative and apoptotic regulation of CG to establish them as potential anticancer agents against prostate, breast, leukemia, lung, liver, colon, and gastric cancers (Table 1 and Table 2).

#### 7.1.1. Prostate Cancer

Prostate cancer is the second most prevalent noncutaneous cancer, next to lung cancer, with the rate of 34.2 per 100,000 individuals [139,140]. According to the statistical reports, prostate cancer is the fifth most common cause of mortality in men, and dominant in North America, Europe, Australia, and New Zealand [140]. McConkey et al. reported that CGs, including oleandrin, ouabain, and digoxin, are potential inducers of apoptosis, mediated by the early release of cytochrome c from mitochondria followed by proteolysis and activation of caspase 3 and caspase 8 [83]. Subsequently, Pathak et al. reported that the oleander extract anvirzel and its derivative compound oleandrin exhibit a high cytotoxic effect on the human prostate (PC-3M, and C4-2) cancer cell lines [94]. These CGs induce cellular apoptosis by inhibiting the telomeric DNA length, arresting the cell cycles at the G2/M phase, and promoting DNA damage. Smith et al. reported that both anvirzel and oleandrin could inhibit the release of fibroblast growth factor-2 (FGF-2) from human prostate cancer cell lines (DU145 and PC3) in a dose and time-dependent manner. The non-cytotoxic concentration of oleandrin (0.1 ng/mL) led to 45.7% and 49.9% inhibition of FGF2 export, and anvirzel showed 51.9% and 30.8% inhibition of FGF-2 release from PC3 and DU145 cells, respectively. Johnson et al. reported that CG steroids, including digitoxin and ouabain, selectively induced the apoptosis of prostate cancer cell line PC3 by inhibiting the Na^+^/K^+^ ATPase of the plasma membrane. This led to the inhibition of prostate target genes including HOXb-13, hPSE/PDEF, hepatocyte nuclear factor 3 alpha and apoptotic inhibitor survivin [76].

Yeh et al. [109] screened the anticancer effect of bufalin and cinobufagine on prostate cancer (LNCaP, DU145, and PC3) cell lines. Their study showed that both bufalin and cinobufagine successfully inhibited cell line proliferation in a dose-dependent manner. The bufalin and cinobufagine increased the intracellular Ca^2+^ concentration and induced apoptosis in all cell lines post 24 hrs treatment. These drugs also increased caspase 3 in DU145 cells and caspase 9 in LNCAP cells. On the other hand, Huang et al. [87] demonstrated that ouabain induced the cytotoxic and cytostatic effects in prostate cancer cell line PC-3 cells in a time and dose-dependent manner. The low concentration of ouabain led to an increase in prostate apoptotic response 4 (Par-4) expression and cell growth arrest by sensitizing the antiproliferative/cytotoxic activities. The higher concentration of ouabain leads to time-dependent loss of mitochondrial membrane potential and causes sustained ROS production and extensive apoptosis of PC-3 cells.

#### 7.1.2. Breast Cancer

Breast cancer is the most commonly diagnosed heterogeneous disease, accounting for 30% of female cancers [141]. It is reported as the leading cause of cancer death in females, with 2.3 million new cases and 684,996 deaths worldwide in 2020 [1]. Kometiani et al. [88] showed that non-lethal concentration of ouabain inhibited the proliferation of MDA-MB-435 (human breast cancer) cell line by antagonizing the Na^+^/K^+^ ATPase pump. The non-lethal concentration of ouabain induced the interaction between Src and EGFR and led to ERK1/2 activation. The ouabain upregulated the cell cycle inhibition of p21Clp1 and downregulated p53, resulting in the growth arrest of breast cancer cells.

In 2006, Bielawski et al. [84] documented the cytotoxic effect of digoxin, ouabain, and proscillaridin A on the human breast (MCF-7) cancer cell line. The study demonstrated that proscillaridin A was more effective in antagonizing the cell proliferation of MCF-7 cells compared to digoxin and ouabain. Proscillaridin A may exhibit its cytotoxic effect by inhibiting the catalytic activities of both topoisomerases I and II. In contrast, digoxin and ouabain can inhibit topoisomerase II catalytic activity but cannot impede topoisomerase I even at higher concentrations. The topoisomerase II enzyme plays a role in DNA replication, transcription, and repair mechanism.

Winnicka et al. [131] also evaluated the apoptosis-mediated cell death induced by ouabain, digoxin, and proscillaridin A on estrogen-independent breast (MDA-MB-231) cancer cell line. In concordance to the report of Bielawski et al., proscillaridin A showed more potency in reducing the cell viability compared to ouabain and digoxin. The inhibition of cell proliferation was mediated by apoptotic cell death. The CG induces their cytotoxic effect by elevating intracellular Ca^2+^ and activating the caspase 3, leading to apoptosis. Besides, many CGs like peruvoside, strophanthidin, convallatoxin, oleandrin, and lanatoside C were reported to have an antiproliferative effect against breast cancer cells [15,18,126,127]. Recently, Howard et al. [142] demonstrated that cardiac glycosides showed a synergistic anticancer effect on triple-negative breast cancer cells MDA-MB-231 by inhibiting the expression of eukaryotic translation initiation factor 4A1 (*EIF4A1*) in a C-Myc dependent mechanism.

#### 7.1.3. Lung Cancer

Lung cancer is the common and leading cause of cancer deaths worldwide, and it accounts for about 18% of the overall cancer death [140]. This cancer is prevalent in North America, East Asia, and parts of central and Eastern Europe. The cardenolide drug (UNBS1450) showed an antitumor effect on human cell lung cancer by inducing the nonapoptotic cell death mediated by the reduction of the HSP70 gene at both RNA and protein levels. UNBS140 reduced the level of HSP70 by down regulating the NFAT5/TonEBP gene. The transcriptional activator NFAT5/TonEBP plays a key role in the transcriptional regulation of HSP70 gene. The reduced level of HSP70 led to the induction of lysosomal membrane permeabilization (LMP) mediated cell death [107].

A series of studies demonstrated the cytotoxic effects of the CG compounds lanatoside C, strophanthidin, and peruvoside drug in A549 lung cancer cell line [15,126,127]. The antiproliferative effects of these CGs were mediated by cell cycle arrest, induced apoptosis, and autophagic cell death. CGs were found to arrest the tumor cells at the G2/M phase by dysregulating the checkpoint proteins and cyclin-dependent kinases like CHK1, CHK2, Cyclin D1, and CDK6. They reported cardiotonic steroids-associated cellular apoptosis by downregulating the proto-oncogenes, including c-FOS, C-Myc, and c-JUN. These steroids induced apoptosis at the pathway level by repressing the MAPK signaling pathway mediated by the inhibition of MEK1, MAP24, and SAPK/JNK and altering the Wnt/β-catenin signaling pathway through downregulating GSK3α and β-catenin genes. Besides, these compounds induced autophagic cell death by attenuating the PI3K/AKT/mTOR signaling pathway through the inhibition of mTOR, LC3, Beclin 1, PI3K, and p62 genes [15,126,127]. Recently, Boff et al. [143] demonstrated the cytotoxic effect of semisynthetic C10 and C18 digitoxigenin analogs on H460 lung cancer cells. Both the compounds demonstrated high cytotoxicity as they induced apoptotic activity and reduced the cellular viability of H460 cells. Among these compounds, C10 showed stronger antiproliferative potential compared to C18.

Another cardenolide drug, glucoevatromonoside (GEV), showed high-affinity binding towards the α subunit of Na^+^/K^+^ ATPase and induced caspase-independent non-canonical apoptosis of lung cancer cell line A549 [135]. GEV exhibited an antiproliferative effect by arresting the cells at the G2/M phase and downregulation of cyclin B1 and p53 expression. Besides, GEV was also found to be effective in reducing the viability and migration of H460 cells, and the combination of GEV with other chemotherapeutic drugs including paclitaxel, cisplatin, irinotecan, and etoposide significantly reduced the clonogenic survival and cumulative population doubling (CPD) of H460 cells [125].

#### 7.1.4. Leukemia

Leukemia is the most prevalent childhood cancer, accounting for 40% of all hematological malignancies [140]. It consists of some 30 heterogeneous lymphoid and myeloid malignancies, having different etiologies and treatment pathways [144]. Jing et al. captured the in vitro anticancer efficacy of bufalin against the human leukemia cells, Jing et al. [110], Masuda et al. [132] and Kawazoe et al. [111]. The bufalin compounds of Chinese medicine chan’s showed selective inhibitory effects on the growth of human leukemia (HL-60, ML1, and U937) cell lines. Bufalin induced apoptosis in a dose-dependent manner by antagonizing the topoisomerase activity and arresting the cells at the G2/M phase. Besides, bufalin influenced a differential expression of apoptosis-related genes. Bufalin exerted its antiproliferative effect by inhibiting the C-Myc expression and induced apoptosis by downregulating the Bcl-2 gene.

The bufalin treatment on the serum-starved U937 human leukemia cell line induced the MAP Kinase activity mediated by the transient activation of Ras, Raf-1, MAP Kinase, and MAP Kinase enzymes and sequential transmission of the signal through these enzymes demonstrated in [112]. The activation of MAP Kinase post bufalin treatment led to the inhibition of intracellular cAMP, which resulted in apoptosis induction. In another study, Kawazoe et al., 1999 reported that bufalin exposure of U937 cells elevated the expression of T lymphoma invasion and metastasis including protein 1 (TIAM 1) at both the mRNA and protein levels [111]. Post bufalin exposure, the cells expressing the sense TIAM 1 RNA showed induced DNA fragmentation. In contrast, the cells expressing antisense TIAM 1 RNA exhibited reduced DNA fragmentation. The induced expression of TIAM 1 led to subsequent activation of Rac1, p21 activated kinase (PAK), and JNK and resulted in cellular apoptosis.

The antileukemic activity of the memi-synthetic cardenolide, UNBS1450, was demonstrated in [74], and the compound reduced the cell viability and induced apoptosis at a nanomolar concentration by antagonizing the NF-kB transcriptional activity and triggering the cleavage of procaspases 8, 9, and 3/7. UNBS1450 treatment downregulated the expression of Mcl-1 and induced activation of Bak and Bax, which led to the mitochondrial outer membrane permeabilization and apoptotic cell death.

Among the other drugs, ouabain induced apoptotic chromatin condensation by repressing NF-kB signaling and explicitly targeting the CD34+ CD38—leukemic cell population [89]. A comparatively less studied CG peruvoside was more effective at inducing apoptosis of the primitive myeloid leukemic cell line KG1a [128]. The drug acted as a potent antileukemic agent arresting the leukemic cells at the G2/M stage and induced apoptosis by upregulating the expression of cyclin-dependent kinase inhibitor 1A (CDKN1A) and triggering the activation of caspase 3, 8 and poly-ADP-ribose polymerase (PARP).

#### 7.1.5. Liver Cancer

Liver cancer is the sixth most common cancer worldwide, with a cumulative incidence of 1.6% for males and 0.6% for females and cumulative mortality of 1.5% for males and 0.6% for females [140], making it the fourth leading cause of cancer mortality worldwide, with elevated rates in North America, Western Europe, Asia, and Africa [140]. Zhao et al. reported the role of neriifolin, a CG compound from *Cerbera manghas*, as a potential anticancer molecule against hepatocellular carcinoma [145]. The drug can reduce cell viability by arresting the HepG2 cells at the S and G2/M phases of the cell cycle. Besides, it induced apoptosis by activating the caspases (3, 8, and 9) and triggered the death receptor pathway by upregulating Fas and FasL expression.

The growth inhibitory effect of CG lanatoside C on epithelial-like (Huh-7 and HepG2) and mesenchymal-like (Mahlavu and FOCUS) hepatocellular carcinoma cells was reported in [123]. The lanatoside C showed high cytotoxicity even at nanomolar concentration and induced apoptosis and autophagic cell death in a time and dose-specific manner. It reduced the cell viability by arresting both Huh-7 and Mahlavu liver cancer cells at the G2/M phase. Besides, lanatoside C facilitated the accumulation of ROS in liver cancer cells by activating ERK1/2. The activated ERK1/2 phosphorylated and repressed the GSK 3β, which in turn activated the JNK1 protein. The JNK1 activation led to the extrinsic apoptotic pathway by activating the cleavage of caspase 8, caspase 3, and PRP proteins [123]. Chao et al., 2017 demonstrated that lanatoside C triggered mitochondrial membrane potential loss, as well as nuclear translocation of apoptosis-inducing factor (AIF), and caused caspase-dependent/independent apoptosis in hepatocellular carcinoma cells Hep3B and HA22T [49]. The lanatoside C induced cellular apoptosis by activating the protein kinase C delta (PKCδ) through Thr505 phosphorylation and antagonizing the AKT/mTOR signaling pathway.

The anticancer effect of bufadienolide compound cinobufagine on hepatocellular carcinoma Huh-7 cells has been reported [133]. Cinobufagine induced its anticancer effect by inhibiting the expression of aurora kinase A (AURKA). Aurora kinase A acted as a serine-threonine protein kinase that plays critical role in mitotic spindle formation, centrosomal duplication, and cytokinesis. In p53 mutant Huh-7 cells, the cinobufagine reduced the cell viability and arrested the cells at the G2/M phase by inhibiting the expression of cyclin B1, CDK1, and PCNA [133]. The activation of AURKA led to the activation and phosphorylation of the p73 gene. The activated p73, in turn, triggered the downstream PUMA, Noxa, and p21 genes and subsequently inhibited the phosphorylated MDM2 [146]. The activated PUMA bound to the anti-apoptotic gene Bcl-2 and promoted caspase activation [147]. Noxa regulated the release of apoptogenic proteins from mitochondria, and p21 regulated the cell cycle progression by inhibiting the activity of CDK2/CDK4 [148].

#### 7.1.6. Colon Cancer

Colon cancer is the third most prevalent cancer in males and the second most prevalent cancer in females accounting for 881,000 deaths worldwide. Colorectal cancer is dominant in Australia and New Zealand, with an incidence rate of 35–42/100,000 in males and 24–32/100,000 in females [140]. Kang et al. [124] verified the cytotoxic and cytostatic effect of lanatoside C against colorectal cancer (HCT116 and HT-29) cell lines. The lanatoside C effectively suppressed colorectal cell growth by inducing mitochondrial dysfunction through the loss of mitochondrial membrane potential (MtMP). The loss of MtMP was mediated by the disruption of K+ homeostasis caused due to the inhibition of Na^+^/K^+^ ATPase. Besides, lanatoside C-induced the HCT 116 cells’ radio sensitivity by surpassing the recruitment of the 53BP1 gene to the damaged DNA, which led to the impairing (formation of 53BP1 foci) of the DNA damage response. The radio sensitization of lanatoside C and repression of 53BP1 foci was caused due to the defect in RNF8/RNF168, mediated by the ubiquitination of JMJD2A or H2AX [124].

The antiproliferative and apoptotic properties of convallatoxin against the colorectal cancer cell line HCT116 was reported in [104]. The antiproliferative and apoptotic properties of convallatoxin were independent of the p53. In the absence of p53, convallatoxin can induce PUMA and NOXA, which were target genes of p53. Besides, convallatoxin induced apoptosis by inhibiting the expression of the anti-apoptotic gene Bcl-2. Pan et al. [96] confirmed that CGs including oleandrin, neriifolin, strophanthidin, gitoxigenin, and convallatoxin, inhibited the growth of the human colorectal cancer cells. Among them, oleandrin was the most effective and reduced the cell viability of colorectal cell lines SW480, HCT116, and RKO [96]. Oleandrin induced apoptosis by downregulating the expression of procaspase 3 and 9 and triggering the expression of caspase 3 and 9. Besides, it antagonized the expression of the anti-apoptotic gene Bcl-2, induced cytochrome c, and pro-apoptotic gene Bax regulation.

#### 7.1.7. Pancreatic Cancer

Pancreatic cancer is the seventh most prominent cause of cancer-associated mortality globally, with an overall survival rate of 9% [140]. The infiltrating pancreatic ductal adenocarcinoma is most dominant among the pancreatic cancers, with a >90% occurrence rate [140,149]. A statistical report suggested that in 2018 a total of 466,000 new cases of pancreatic ductal adenocarcinoma were observed worldwide, with an incidence rate of 6.2 per 100,000 individuals in developed countries and 1.5 per 100,000 in developing countries for both males and females [140]. More than 90% of the pancreatic cancer cases are sporadic; while smoking, obesity, and long-standing type 2 diabetes are regarded as major risk factors [150]. Newman et al. [97] reported lipid soluble CG oleandrin as a potent inhibitor of human pancreatic cell PANC-1. The oleandrin inhibited cell proliferation by arresting the cells at the G2/M phase and induced autophagy rather than apoptosis, which led to mitochondrial condensation, followed by the mitochondria relocation to a perinuclear location accompanied by cellular vacuolation. This is followed by drug-induced autophagy by upregulating the expression of the microtubule-associated proteins 1A/1B light chain 3B (LC3II) gene. The LC3 gene in conjugation with the ATG8 gene regulates the autophagosome’s elongation and maturation [151]. Oleandrin exhibited its antiproliferative effect on PANC-1 cells by inhibiting the expression of PAK-1 and upregulating the expression of PERK in concentration-dependent manner, which in turn altered the AKT/mTOR/p70S6K pathway [97].

The cardiotonic drug bufalin potentiated the antitumor efficacy of gemcitabine against the most popularly used pancreatic cancer Bxpc-3, Mia PaCa-2, and Panc-1cell lines. Bufalin induced the apoptotic ability of gemcitabine by inhibiting the expression of Bcl-2 and activating the cleavage of caspase-3 [114]. The bufalin-mediated apoptosis was also accompanied by the upregulation of apoptosis signal-regulating kinase 1 (ASK1). ASK1 acts as a ROS-sensitive protein, which regulates the activation of AP1, Rac1, cdc2 Kinase, and JNK and plays a crucial role in stress apoptosis [152]. Besides, the activation of JNK has a fundamental role in an inflammatory response, cytokine production, and cellular apoptosis. Li et al. reported that bufalin suppressed the proliferation of pancreatic cancer (PANC-1 and CFPAC-1) cells in a dose and time-dependent manner [115]. The steroid reduced the cell viability by arresting the cells at the G2/M phase of the cell cycle, mediated by the inhibition of the cyclin B1/CDK1 expression. It induced apoptosis by inhibiting the expression of the anti-apoptotic gene HSP27 and its partnering molecule P-AKT. Bufalin played a crucial role in activating procaspase-3 and procaspase-9. Moreover, it inhibited Bcl-2 expression and induced Bax/Bcl-2 ratio [115].

A few more studies conducted by Liu et al. demonstrated that bufalin showed antitumor activity on the pancreatic cells BxPc3 and Sw1990 by arresting the S phase cells [116]. Bufalin reduced the cell viability by inhibiting cyclin D1 and cyclin E1, which induced the expression of the P27 gene and regulated the transition of the cell cycle S-G2 phase. In addition, bufalin exerted its antiproliferative effect by significantly reducing the expression of cell cycle regulator C-Myc and its downstream transcription factor NF-kB. The transcription factor C-Myc plays a key role in regulating cell proliferation, cell differentiation, and apoptosis [153]. Besides, it also supports tumor progression and metastasis [154]. The C-Myc was documented as a significant oncogene for pancreatic cancers and correlated with the perineural invasion [155]. Hence, the inhibition of the C-Myc and its downstream NF-kB transcription factor makes bufalin a potential drug for pancreatic cancer therapy.

### 7.2. In Vivo Studies on the Anticancer Properties of Cardiac Glycosides

The CG compound digitoxin could inhibit the two-stage carcinogenesis of mouse skin papillomas induced by 7,12-dimethylbenz [a] anthracene [DMBA] and 12-O-tetradecanoylphorbol-13-acetate [TPA], and mouse pulmonary tumors triggered by 4-nitroquinoline-N-Oxide (4NQO) and glycerol [156]. Digitoxin treatment led to a 20% reduction in the rate of skin papilloma-bearing mice at 20 weeks and a 50% reduction in the quality of pulmonary tumor-bearing mice after 25 weeks. Afaq et al. reported the antitumor effect of oleandrin on 12-O-tetradecanoylphorbol-13-acetate (TPA)-induced CD1 mouse skin tumor model and showed inhibition of tumor promotion markers [157]. The oleandrin exposure significantly inhibited the TPA-mediated cutaneous edema, hyperplasia, epidermal ornithine decarboxylase (ODC), and cyclooxygenase-2 (COX-2) protein expression. The oleandrin treatment also resulted in TPA reduction inducing the expression of PI3K, phosphorylation of AKT, and NF-kB activation. ODC acted as a rate-limiting enzyme that played a significant role in regulating cell proliferation, tumor promotion, and cancer [158]. The cyclooxygenase-2 enzyme helped cutaneous inflammation, cell proliferation, and skin tumor promotion [159]. Hence, the inhibition of ODC and COX -2 was essential to reduce the inflammation and exert the antitumor effect. The PI3K/AKT was reported as a critical regulatory molecule regulating growth of cells and malignant transformation. AKT, a serine threonine protein kinase, acts as a downstream substrate of PI3K [160]. The PI3K/AKT signaling regulates cell survival by triggering the NF-kB pathway.

That cardenolide digitoxin could inhibit tumor growth in mice grafted with neuroblastoma cell lines SH-SY5Y and Neuro-2a [77]. The digitoxin treatment significantly reduced the SH-SY5Y grafts by 44% and Neuro-2a grafts by 19%. The study hypothesized that the digitoxin mediated inhibition of the neuroblastoma grafts might be due to induction of apoptosis resulting from the elevated level of intracellular Ca^2+^ ion and activation of the caspase cascade. Besides, the cardiotonic drug effectively inhibited the FGF-2 stimulated angiogenesis in the chick chorioallantoic membrane (CAM) assay. Han et al. orthotopically transplanted human hepatocellular carcinoma cells in BALB/Cnu/nu nude mice and reported that bufalin at a non-toxic concentration exhibited antitumor property in the in vivo model [161]. Bufalin led to the induction of apoptosis by inhibiting Bcl-2 and Bax expression.

## 8. Molecular Targets of Cardiac Glycosides in Cancer Therapy

Ouabain plays a key role in activating ROCK I and ROCK II genes in HeLa cells [91]. ROCK II and I are effector genes. Their activation triggers the membrane blebbing and induces apoptosis within the cell line [91]. Trenti et al. demonstrated the anticancer effect of ouabain on A549 and H1975 cell lines [90]. The ouabain reduced the cell proliferation and triggered apoptosis by downregulating the level of Bcl-2 gene mediated by the activation of JNK and acts as a key player in regulating cell proliferation, differentiation, and apoptosis. Among the JNK genes, JNK1 phosphorylates Bcl-2, causing the inhibitory interaction between Bcl-2 and Beclin 1 [162,163]. The dissociation of Bcl-2 from Beclin 1 leads to the inactivation and degradation of Bcl-2 [90]. The ouabain treatment inhibited the EGF-induced activation of ERK ½ and Akt signaling in human medulloblastoma (DAOY) cells [92]. Besides, the cardiotonic drug attenuated the formation of EGF-induced actin stress fibers and antagonized DAOY cells motility by triggering the stress signaling response. The recent, reports on ouabain exerts its cytotoxic effects against various cancer cells by antagonizing the expression of STAT3 [164].

The inhibitory effect of digoxin on HeLa cells at the post-transcriptional level was reported in [86]. The digoxin exposure led to the downregulation of the SRSF3 gene. The depletion of SRSF3 altered the cell cycle specific genes, including cyclin D1, cyclin B1, and H3P, which disrupted the cell cycle progression at the G2/M phase and induced cellular apoptosis. Besides, SRSF3 is a proto-oncogene, which plays a key role in regulating cell proliferation and tumor induction [165]. The expression of SRSF3 was reported to be associated with the development of epithelial ovarian cancer malignancy. Hence the downregulation of the gene is key to attenuate cancer cell growth [166]. Lee et al. demonstrated that the CG drug digitoxin suppressed the intracellular accumulation of HIF 1α during the hypoxic condition in human glioma stem cells [17]. Besides, digitoxin effectively attenuated the hypoxia-induced VEGF expression and ERK½ signaling pathway. VEGF is a downstream target gene of HIF 1α, which plays a role in the formation of tumor angiogenesis [167]. Besides, digitoxin suppressed hypoxia induced cellular invasion and migratory activities by antagonizing the expression of migration related proteins, including MMP-2, MMP-9, uPA, and p-ERK.

The cytotoxic effect of oleandrin on the proliferation, morphology, and apoptosis of human osteosarcoma (U2OS and SaOS-2) cells was reported in [98]. Oleandrin treatment inhibited the proliferation and invasion of osteosarcoma cells and induced cellular apoptosis by repressing the Wnt/β-catenin signaling pathway. Oleandrin significantly downregulated the expression of Wnt target genes, including C-Myc, survivin, cyclin D1, MMP-2, and MMP-9 at both mRNA and protein levels. Besides, it also antagonized the expression of total β-catenin and reduced its nuclear localization [98]. Wang et al. reported the anticancer effect of bufalin against the MCF-7 breast cancer cell line [120]. Bufalin acted as a potential small-molecule inhibitor and inhibited the activity of steroid receptor co-activators, including SRC-1 and SRC-3. These co-activators played a role in regulating cancer cell proliferation, invasion, and metastasis [120]. Kang et al. reported that bufalin induced cellular apoptosis in NSCLC cell line H1975 by downregulating the expression of anti-apoptotic Bcl-2 family member protein Mcl-1 [118]. Bufalin treatment triggers the proteosomal degradation of Mcl-1 mediated by the activation of GSK-3β.

The cardiac glycoside digitoxin inhibits cellular growth and triggers apoptosis in the human renal cancer (TK-10) cell line, breast cancer (MCF-7) cell line and melanoma (UACC-62) cell line by inducing the level of DNA topoisomerase II cleavable complex [79]. Later in 2007 Lazaro discussed the mechanism related to the anticancer properties of digitoxin [80]. The digitoxin treatment resulted in the downregulation of intracellular levels of K+ and upregulation of intracellular levels of Na^+^ and Ca^2+^ ions. The increased level of intracellular Ca^2+^ ions induced apoptosis of the cancer cells. Besides, digitoxin exerts its anticancer activity by antagonizing the expression of IL8, an essential gene associated with several human cancers, including acute myelogenous leukemia, B cell chronic lymphocytic leukemia, brain tumor, breast cancer, and colon cancer [80,81]. Parallelly, the digitoxin and its structurally related CGs suppress the TNFα/NF-kB signaling pathway activation. The repression of the pathway leads to the induction of apoptosis. Besides, digitoxin induced cytotoxicity in leukemia cells by triggering the activation of ERK ½, which generates the levels of cell cycle inhibitor p21Clp1 [80,82].

Wang et al. demonstrated that both digitoxin and ouabain downregulated the expression of p53 in lung cancer (A549 and H1355) cell lines by reducing its protein level synthesis [78]. The degradation of p53 in these cancer cells was triggered by the activation of the Src/MAPK signaling pathway as the digitoxin and ouabain treatment blocked Src or MAP/ERK kinase inhibitors. Zhang et al. reported that CGs including digoxin, ouabain, and proscillaridin A played a key role in suppressing the HIF-1α protein synthesis and expression of HIF-1 target genes in several cancer cells [45]. The overexpression of HIF-1α induces mortality in patients suffering from the bladder, brain, breast, cervix, lung skin, and stomach cancers. The exposure of Hep3B cells to ouabain and proscillaridin A inhibited the hypoxia-induced expression of both HIF-1α and HIF-2α proteins in a dose-dependent manner. The digoxin treatment inhibited the expression of HIF-1α protein and HIF-1 target genes VEGF, GLUT1, HK1, and HK2. Lin et al. reported that the CG digoxin played a crucial role in suppressing the proliferation, invasion, migration, and colony formation in A549 lung cancer cells by antagonizing the Src-related signaling pathway [85]. The study delineated that digoxin treatment attenuated the Src activity and downregulated the EGFR and STAT3 activities.

The oleandrin exerts anti-carcinogenic, anti-inflammatory, and growth-modulatory effects on human cancer cells inhibiting the ceramide induced NF-kB and AP-1 activation [99] and ceramide acts as a key secondary messenger for the transcription factors, including NF-kB and AP-1, related to inflammation and apoptosis. Hence, the suppression of these transcription factors is vital to block tumorigenesis and inflammation. The anticancer effect of ouabain on the glioma U-87 MG cells was reported in [93]. Ouabain treatment suppressed cell survival, growth, and migration by downregulating the expression of pAkt, mTOR, pmTOR, and HIF1α in a dose-dependent manner. Berges et al. denoted that the CG drug proscillaridin A exerted anticancer activity against glioblastoma cells in a microtubule-dependent mechanism [168]. The proscillaridin A exposure led to the microtubule dynamic instability in glioblastoma cells resulting from the GSK3β activation and EB1 phosphorylation, which led to inhibition of cell migration. The antiproliferative effect of proscillaridin A on the prostate cancer cell lines LNCaP and DU145 was reported in [100]. The CG drug inhibited cell proliferation and induced apoptosis in a dose-dependent manner. Among these cells, LNCaP was more sensitive to proscillaridin A compared to DU145. Proscillaridin A treatment suppressed the Bcl-2/Bax ratio and induced the activity of Caspase-3 and cleavage of PARP. Besides, the drug was found to inhibit JAK2/STAT3 signaling and induce doxorubicin toxicity in prostate cancer cells.

Costa et al., 2019 identified the antitumor activity of proscillaridin A against C-Myc driven leukemic cells [102]. Proscillaridin A treatment downregulated the C-Myc mRNA and simultaneously upregulated the T-cell activation and differentiation markers NOTCH3, HES1, TCR, and CD3 expressions. Besides, proscillaridin A therapy led to reduced lysine acetylation at H3K9, H3K14, H3K18, H3K27 and resulted in a loss of histone H3 acetylation in a time dependent manner. Li et al., 2018 reported the cytotoxic effect of proscillaridin A against NSCLC cells. Additionally, proscillaridin A inhibited cell proliferation and triggered apoptosis by increasing the level of intracellular Ca^2+^, activating the AMPK pathway, and antagonizing ACC and mTOR [101]. The proscillaridin A treatment led to the upregulation of death receptor four and downregulation of NF-kB. The anticancer activity of proscillaridin A against the osteosarcoma cell line 143B cells. Besides that, proscillaridin A treatment also reduced cell proliferation, inhibited cellular metastasis, and induced apoptosis by downregulating the expression of Bcl-xl and MMP2 at both mRNA and protein levels [103].

The anticancer effect of convallatoxin, which acts as a dual inducer of apoptosis and autophagy was reported in [105]. The convallatoxin treatment induced apoptosis in HeLa cells by triggering cleavage of caspase-3 and PARP. The cardiotonic drug was found to induce autophagy in a dose and time-dependent manner by inhibiting the (mTOR)/p70S6K signaling pathway. Besides, convallatoxin acts as an antiangiogenic compound by inhibiting the human umbilical vein endothelial cells (HUVEC) in a dose dependent manner. The anticancer effect of convallatoxin on breast cancer was reported in [106]. Convallatoxin exerted its cytotoxic effect on MCF-7 cells by arresting the cells at G0/G1 phase and reducing the viability of MCF-7 mammospheres (MMs). The convallatoxin treatment modulated the epithelial–mesenchymal transition (EMT) pathway by inhibiting the EMT markers, including EGFR, pEGFR, β-catenin, Vimentin, and Slug. Besides, it induced apoptosis by triggering the pro-apoptotic proteins PARP and phospho-p53 and inhibiting the anti-apoptotic proteins Bcl2 and XIAP [106]. The human NSCLC cell line A549 exhibits NF-kB mediated cytoprotective effects [108]. Mijatovic et al. demonstrated that the cardenolide drug UNBS1450 treatment inhibited the DNA binding capacity of p65 and deactivated NF-kB transcriptional activity to diminish the cytoprotective effect.

The cytotoxic effect of bufalin on the human leukemia cell line HL-60 was reported in [113]. Bufalin exhibited the anticancer effect by markedly decreasing the expression of topoisomerase IIα and IIβ in a dose-dependent manner that triggered cellular apoptosis. Kawazoe et al. showed that bufalin induced apoptosis in leukemia U937 cells by upregulating the expression of Tiam1 at both mRNA and protein levels [111]. The activated Tiam1 stimulates the JNK pathway by triggering the activation of Rac1 and PAK. Zhang et al. reported the anticancer activity of bufalin against the osteosarcoma cells U-2OS and Saos-2 [121]. The bufalin treatment inhibited the cell proliferation in a dose-dependent manner and induced the mitochondria dependent apoptosis by triggering the intracellular reactive oxygen species production and downregulating the expression of miR-221. Bcl-2 binding component 3 (BBC3), also known as the p53 upregulated modulator of apoptosis (PUMA), is a potential target of miR-221. BBC3 regulates the mitochondrial apoptotic pathway by maintaining the integrity of the mitochondrial outer membrane [169].

The cancer cells depend on the EMT for their metastasis and invasive properties [170]. TGF-β plays a role in inducing EMT and supporting metastasis of several cancers [171,172,173]. Zhao et al. reported that bufalin treatment efficiently suppressed the TGF-β induced EMT and migration in A549 cells [119]. Besides, the study showed that bufalin treatment inhibited the TGF-β induced upregulation of Twist-2 and zinc finger E-box binding homeobox 2 (ZEB2) and phosphorylation of Smad2 and Smad3. The transcription factors Twist-2 and ZEB-2 repress E-cadherin expression and regulate the EMT and migration in cancer cells [119,174]. Moreover, the Smad signaling components, Smad2 and Smad3 also support the TGF-β triggered EMT [175]. Previous studies have suggested that the TGF-β receptor I (TβRI) phosphorylates both Smad2 and Smad3, while TβRII phosphorylates TβRI [176]. Upon treatment with bufalin both TβRI and TβRII downregulated the expression, which inhibited the phosphorylation of Smad2 and Smad3. Wang et al., 2018 reported that bufalin treatment inhibited gastric cancer cell proliferation and colony formation in a dose-dependent manner [122]. Bufalin markedly suppressed the epithelial mesenchymal transition (EMT) and migration in gastric cancer by inhibiting the transcription factor achaetescute like 2 (ASCL2) and antagonizing the expression of invasion-related genes like MMP2, MMP9, and vimentin. The list of CGs and their molecular targets in various cancer cells is documented in Table 1 and chemical structures in Figure S1A–L.

## 9. Molecular Docking Studies on Cardiac Glycosides

Several studies in the recent past have established the interaction of cardiac glycosides and their target receptors through molecular docking. The docking information was useful in identifying the site of CGs drug action and monitoring their pharmaceutical effects. Paula et al. reported the computational docking of digoxin with monoclonal antibody 1B3 [177]. The lactone and steroid moieties of digoxin were deeply embedded in a narrow, close binding pocket of mAB 1B3 through hydrophobic interaction, whereas the γ- digitoxose group was partially solvent-exposed and formed a hydrogen bond with the amino acids residing in the binding pockets of mAB 1B3. Tian et al., through the Induced Fit Docking (IFD) method, reported the molecular docking of bufalin and its unsaturated derivatives Δ^8,14^-anhydrobufalin and Δ^14,15^-anhydrobufalin with the androgen receptor. Their study revealed that the lactone ring of Δ^8,14^-anhydrobufalin was in close proximity and formed bifurcated H-bond from the C-24 group with ARG 752 and GLN 711. Besides, the hydroxyl group of C-3 acted as the hydrogen donor and formed a third H-bond with THR 877 to support the ligand binding and receptor activation. Further, the docking study revealed that Δ^14,15^-anhydrobufalin lacked the hydrogen bond with ARG 752, and bufalin lacked the H-bond between the hydroxyl group of C-3 and THR 877. The ligand conformations denoted that Δ^8,14^-anhydrobufalin was more bent and formed arched shape than bufalin and Δ^14,15^-anhydrobufalin, which made Δ^8,14^-anhydrobufalin more favorable for the ligand-binding domain of androgen receptor. The study confirmed that among these three compounds, Δ^8,14^-anhydrobufalin has a more potent binding affinity towards androgen receptors [178].

Magpusao et al. established the interaction of ouabain and its analogs with Na^+^/K^+^ ATPase through molecular docking and correlated the binding energy and root-mean square-deviation (RMSD) of the docked compound with in-vitro experimental data obtained from Na^+^/K^+^ ATPase inhibition and MDA-MB-231 breast cancer cell migration assay [179]. The ouabain binding site in the α subunit of Na^+^/K^+^ ATPase is located in the extracellular cleft between transmembrane segments αM1 and αM6. The ouabain can be present in two orientations (i) the lactone ring facing the cytoplasmic side and (ii) the lactone ring facing the extracellular side. Among the ouabain analogs, analogs 2 and 3 were more potent, and they adapted similar orientation to the crystal structure of ouabain bound to Na^+^/K^+^ ATPase. The hydrophobic residues, including I315, F316, F783, and F786, stabilized the nonpolar and concave α-surface of ouabain and its analogs 2 and 3. In contrast, the polar β-surface of these compounds was stabilized by H-bond formation between C-19 OH and C-14 OH of the CG with N111 and T797 of αM1 and αM6, respectively [179]. The binding energy for analogs 2 and 3 obtained from the docking study was significantly correlated with the IC_50_ values for Na^+^/K^+^ ATPase and cell migration inhibition. Later, Schneider et al. assessed the cytotoxicity of CG, glucoevatromonoside (GEV) by computational docking [135]. The docking analysis suggested that GEV was located in the extracellular ion pathway cavity of the α subunit of the Na^+^/K^+^ ATPase. The lactone ring of GEV was embedded in the deeper side of the cavity, and the steroid core was found to interact with the transmembrane helices αM1-6. The α-surface of the steroid cores were oriented towards the hydrophobic side chains of αM4-6, and the β-surface of the steroid cores were located in close proximity to the polar side chains of αM1-2. The disaccharide of GEV was found to interact with the αM7-8 loop of the transmembrane helices [135]. Recently, Reddy et al. through molecular docking analysis, identified the binding sites of lanatoside C with key signaling proteins associated with both cell survival and cell death and confirmed the ability of the CG drug to inhibit various cancer targets [15]. Among the tumor-associated proteins, AKT formed three hydrogen bonds with lanatoside C at residues GLU 323, ASP324, and ASP326, PI3K formed seven hydrogen bonds at residues ILE703, ALA704, SER706, ARG707, SER ILE703, SER706, ARG707, SER753, ALA704, LYS809, and LYS807 and mTOR formed five hydrogen bonds at residues ASP2360, VAL2364, ASP2433, THR2434, and THR2436 [15].

## 10. Clinical Trials of Cardiac Glycosides in Cancer Treatment

For decades CGs were known for their role in treating cardiovascular diseases. The cardiac glycoside-containing extracts of *Nerium oleander* such as anvirzel and PBI-05204 have recently shown promising results in phase I and II clinical studies for treating solid cancers. Mekhail et al. in their phase I clinical trial of anvirzel, reported the maximum tolerated dose (MTD) and safety of the drug in patients with advanced solid refractory tumors. Among the 18 patients enrolled in the study, 78% of them showed mild injection site pain. The other toxicities involved were fatigue, dyspnea, and nausea. According to the phase I clinical trial, the safe dose for anvirzel was defined as <1.2 mL/m^2^/day [180]. Besides the safety profile, the antineoplastic potential of anvirzel was also evaluated in combination with carboplatin and docetaxel in phase I clinical trial on NSCLC patients [181]. PBI-02504, an extract from *Nerium oleander*, was evaluated in phase I clinical trial on patients with advanced solid tumors. The PBI-05204 drug contains oleandrin as an active principle and plays a key role in attenuating the mTOR pathway [181]. Forty-six patients were treated with PBI-05204 with adenocarcinoma and colon carcinoma. The PBI-05204 drug was well tolerated up to a dose of 0.2255 mg/kg per day. The common drug-related adverse effects were observed as fatigue (56.5%), nausea (41.3%), and diarrhea (32.6%) [182]. The PBI-05204 treatment showed significant tumor regression in colorectal, bladder, and fallopian tube cancer patients. Recently, Roth et al. in their phase II clinical trial, evaluated the anticancer efficacy and safety of PBI-05204 in patients with stage IV metastatic pancreatic ductal adenocarcinoma (https://clinicaltrials.gov/ct2/show/NCT02329717, accessed on 30 March 2021). Among the 44 patients enrolled in the study, only ten patients survived for >4.5 months with a median PFS of 56 days. The grade 3 treatment-related adverse events were fatigue, vomiting, nausea, decreased appetite, and diarrhea. The study denoted that PBI-05204 was well tolerated with low-grade gastrointestinal side effects. The CG drug showed significant results against glioblastoma when combined with chemotherapy and radiotherapy [183]. Currently, 26 clinical trials of digoxin on cancer patients are underway, including eleven successfully completed trials. (https://clinicaltrials.gov/ct2/results?cond=cancer&term=digoxin, accessed on 30 March 2021). The combination of digoxin with cisplatin showed significant therapeutic improvement over the conventional chemotherapeutics for the patients with advanced head and neck carcinomas (https://clinicaltrials.gov/ct2/show/NCT02906800, accessed on 30 March 2021). Treatment with digoxin induced the efficacy of cisplatin-based chemotherapy by inducing anti-tumor immunity. Frankel et al. in a phase I study, evaluated the anticancer efficacy of digoxin in combination with trametinib (MEK inhibitor) to treat patients with BRAF melanomas [184]. Among the 20 patients evaluated, 14 patients were NRAS wild type, and six patients were NRAS mutant. The drug related adverse events were observed as rash, diarrhea, nausea, and fatigue. Combining digoxin and trametinib induced the response in 20% of patients and controlled the disease in 65% of patients. The digoxin was found to synergize with MEK inhibitor and showed a better disease control rate (83%) for the patients with NRAS mutant metastatic melanoma.

## 11. Novel Aspects of Cardiac Glycoside Research

Artificial intelligence (AI) and machine learning (ML) have emerged as essential tools for developing novel drug candidates and could be key players for efficient computational searches over trillions of biologically active compounds [185]. Moreover, ML and its components have been publicized for its rapid and efficient diagnosis, understanding disease dynamics, and repositioning old drugs. Drug repositioning offers many advantages over rational drug discovery, especially to palliate the conventional usage and also to delimit the failure rates in clinical trials [186]. Scientists have proven that geometric deep learning may assist in forecasting and creating molecular surface interaction fingerprinting to study biomolecular interactions and assist in CG interaction fingerprint with target proteins. AtomNet is a neural network based on millions of scientifically pre-determined small chemicals binding affinity measurements and thousands of protein structures to forecast binders [179,180]. Mounting evidence suggests the prospective role of CGs in inhibiting malignant tumor cell proliferation and apoptotic induction in various cancers [187]. More quick and efficient discovery of CGs targeting critical oncogenic pathways will rely on molecular structure-based screening techniques, high throughput screening and the application of deep learning networks. Besides anticancer potential, CGs also possess antiviral activity. Cardiac glycosides including digitoxin, digoxin, ouabain, and their convolution have a profound influence in inhibiting viral proliferation and may be effective in virus-associated tumors. CGs intermediates and their mode of action as immune modulators [188] and other cytotoxic activities [134] have also been reported and need further evaluation. A highly specialized targeted deep ML tool could probably address the question of identifying potential candidates within the available CG database.

## 12. Conclusions and Future Prospectus

This review focused and highlighted the therapeutic effect of natural compounds, particularly CGs, to stabilize the altered gene functions in cancer. We emphasized the significance of CGs as potential inhibitors of several oncogenic genes and transcription factors involved in cancer signaling pathways. In clinical trials, digoxin, strophanthidin, and ouabain were found to be successful against several tumor-causing TFs such as HIF-α, NF-Kb, AP-1, and C-Myc, demonstrating the high selectivity of CGs against transcription alteration. The recent publications on CGs and nuclear factors are also reviewed, which motivates important directions for future studies. Therefore, this class of bio compounds represents an alternative to chemical and synthetic drugs associated with severe side effects. Its emerging importance and acceptance in targeted TF therapy in cancer to develop agents that can suppress the elusive TFs dependency in cancer was also highlighted.

Recent advancements in functional genomics combined with sensitive analytical methodologies such as mass spectroscopy to detect TFs, are now providing extensive details on TF involvement across diverse forms of cancers. Single-cell transcriptomic and proteomic data sets with high resolution may aid in generating reliable information on the effect of CGs on cancer versus normal cells in various tumors. Furthermore, high throughput screening of CGs using tumoroids and organoids may supplement preclinical effectiveness and safety research. These technologies are expected to unravel the regulatory mechanisms that orchestrate the transcriptional processes regulating tumorigenesis. Among the large number of dysregulated oncogenic TFs, only a few have been successfully identified and targeted. These findings in coordination with gene-specific therapy could be the new paradigm for targeted cancer therapeutics. Thus, a comprehensive understanding of the transcriptional network proteins could potentially decipher the TFs misregulation in cancer. Recent improvements in cryo-electron microscopy (cryoEM) have also opened up new avenues for understanding the structures of CG-protein interaction dynamics, which was difficult to dissect using traditional structural biology techniques.

Future studies could focus on discovering novel TFs and pathways involved in the complex transcriptional network regulating tumorigenesis. Besides, improved algorithms and models for network analysis would substantially increase the probability of finding druggable TF targets. Bioprospecting of novel CGs and extensive studies may also uncover novel pathways to inhibit dysregulated TFs.

## Figures and Tables

**Table 1:** List of Cardiac glycosides and their molecular targets in various cancer cells (Figure S1A–L).

Cardiac Glycosides and Referred Chemical Structure	Cancer Types	Molecular Targets	References
digitoxin (Figure S1A)	Prostate	Inhibits HOXb—13, hPSE/PDEF, hepatocycte nuclear factor 3α, and survivin	[76]
	Neuroblastoma	Increases intracellular Ca^2+^ ion and activates caspase cascade	[77]
	Lung	Down-regulates cyclin B1, cdc2, and survivin	[54]
		Down-regulates p53, p21, p27, and checkpoint kinases Chk1 and Chk2	
		Triggers Apo2L/TRAIL-induced apoptosis and up-regulates the expression of death receptors 4 and 5	[71]
		Activates Src/MAPK signaling pathway	[78]
	Glioblastoma	Attenuates the hypoxia-induced VEGF expression and ERK ½ signaling pathway	[17]
		Antagonizes the expression of migration-related proteins like MMP-2, MMP-9, uPA, and p-ERK	
	Renal and melanoma	Induces DNA topoisomerase II cleavable complex	[79]
	Breast and colon	Antagonizes the expression of IL8	[80,81]
	Leukemia	Activation of ERK ½ and inhibition p21^Clp1^	[82]
digoxin (Figure S1B)	Prostate	Mitochondrial Cytochrome c release and activates caspase 3 and caspase 8	[83]
	Breast	Inhibits the catalytic activity of topoisomerase II	[84]
	Lung	Triggers Apo2L/TRAIL-induced apoptosis and up-regulates the expression of death receptors 4 and 5	[71]
		Inhibits the Src-related signaling pathway and down-regulates EGFR and STAT3 activity	[85]
	Cervical	Down-regulates SRSF3	[86]
	Liver	Inhibits the HIF-1α protein synthesis and expression of HIF-1 target genes	[45]
ouabain (Figure S1C)	Prostate	Releases cytochrome c from mitochondria and activates caspase 3 and caspase 8	[83]
		Inhibits HOXb—13, hPSE/PDEF, hepatocycte nuclear factor 3α, and survivin	[76]
		Results in loss of mitochondrial membrane potential and sustains reactive oxygen species (ROS) production	[87]
	Breast	Degradation of Na/K-ATPase mediated by endocytosis and induces expression of p21^Clp1^	[61]
		Induces the interaction between Src and EGFR and leads to the activation of ERK ½	[88]
		Inhibits the catalytic activity of topoisomerase II	[84]
	Leukemia	Represses NF-KB signaling	[89]
	Lung	Triggers TRAIL-induced apoptosis and down-regulates the expression of Mcl-1	[72]
		Down-regulates Bcl-2 and activates JNK	[90]
		Down-regulates the expression of p53 and activates the Src/MAPK signaling pathway	[78]
	Neuroblastoma	Stimulates the phosphorylation of Erk1/2, Akt, and Bad and down-regulates the expression of Bcl-XL and Bcl-2	[73]
	Cervical	Activates ROCK I and ROCK II effector genes	[91]
	Brain	Inhibits the EGF-induced activation of ERK ½ and Akt signaling pathway	[92]
		Down-regulates the expression of p-Akt, mTOR, p-mTOR, and HIF-1α	[93]
	Liver	Inhibits the hypoxia-induced expression of both HIF-1α and HIF-2α proteins	[45]
oleandrin (Figure S1D)	Prostate	Releases cytochrome c from mitochondria and activates caspase 3 and caspase 8	[83]
		Inhibits the telomeric DNA length and promotes DNA fragmentation	[94]
		Inhibits FGF2 export	[95]
	Colon	Down-regulates the expression of procaspase-3 and -9, Inhibit the expression of Bcl-2, and induces the expression of cytochrome C and Bax	[96]
	Pancreatic	Induces autophagy by upregulating the expression of LC3II	[97]
		Inhibits the expression of PAK-1 and up-regulate the expression of PERK	
	Lymphoma	Suppresses of TNF-induced NF-kB activation and blocks AP-1, JNK, and MEK	[48]
	Lung	Triggers Apo2L/TRAIL-induced apoptosis and up-regulates the expression of death receptors 4 and 5	[71]
	Osteosarcoma	Represses the Wnt/β-catenin signaling pathway	[98]
	Cervical & Breast	Inhibits ceramide-induced NF-kB and AP-1 activation	[99]
proscillaridin A (Figure S1E)	Prostate	Suppresses the Bcl-2/Bax ratio and JAK2/STAT3 signaling	[100]
	Lung	Induces level of intracellular Ca^2+^, activate AMPK pathway, and antagonizes ACC and mTOR	[101]
	Leukemia	Down-regulates myc mRNA and up-regulate the T-cell activation and differentiation markers NOTCH3, HES1, TCR, and CD3	[102]
	Osteosarcoma	Down-regulates the expression of Bcl-xl and MMP2	[103]
convallatoxin (Figure S1F)	Colon	p53 independent apoptosis by inducing the expression of p53 target gene PUMA and NOXA	[104]
	Cervical	Triggers cleavage of caspase-3 and PARP and inhibits the (mTOR)/p70S6K signaling pathway	[105]
	Breast	Arrests the cells at G0/G1 phase and modulates the EMT pathway	[106]
UNBS1450 (Figure S1G)	Lung	Inhibits the expression of HSP70 gene	[107]
		Inhibits the DNA binding capacity of p65 and NF-kB mediated cytoprotective effects	[108]
	Leukemia	Antagonizes the NF-kB transcriptional activity and triggers the cleavage of procaspases 8, 9, and 3/7	[74]
bufalin (Figure S1H)	Prostate	Increases the intracellular Ca^2+^ concentration and induces apoptosis by triggering caspase 3 and caspase 9	[109]
	Leukemia	Arrests the cell cycle at the G2/M phase and antagonizes topoisomerase activity	[110]
		Induces the expression of TIAM 1	[111]
		Induces the MAP Kinase activity by the transient activation of Ras, Raf-1, MAP Kinase Kinase, and MAP Kinase enzymes	[112]
		Inhibits expression of topoisomerase II_α_ and II_β_	[113]
	Pancreatic	Inhibits the expression of Bcl-2 and up-regulates (ASK1)/JNK	[114]
		Inhibits the expression of anti-apoptotic gene HSP27 and its partnering molecule P-AKT	[115]
		Inhibits expression of c-Myc and its downstream TF NF-kB	[116]
	Melanoma	Arrests at the G2/M phase	[117]
	Lung	Induces the expression of p53 and p21(WAF1/CIP1) and inhibits the expression of Cyclin D1	[53]
		Triggers Apo2L/TRAIL-induced apoptosis and up-regulates the expression of death receptors 4 and 5	[71]
		Leads to the proteosomal degradation of Mcl-1	[118]
		Inhibits TGF-β induced upregulation of Twist-2 and ZEB2 and phosphorylation of Smad2 and Smad3	[119]
	Breast	Inhibits the activity of steroid receptor co-activators, SRC-1 and SRC-3	[120]
	Osteosarcoma	Triggers the intracellular ROS production and down-regulates the expression of miR-221	[121]
	Gastric	Inhibits the transcription factor ASCL2 and antagonizes the expression of invasion related genes like MMP2, MMP9, and Vimentin	[122]
lanatoside C (Figure S1I)	Breast & Lung	Dysregulates the checkpoint proteins and cyclin-dependent kinases like CHK1, CHK2, Cyclin D1, and CDK6 and down-regulates the proto-oncogenes, including c-FOS, c-MYC, and c-JUN	[15]
	Liver	Facilitates the accumulation of ROS by activating ERK1/2	[123]
		Activates PKC δ and antagonizes the AKT/mTOR signaling pathway	[49]
glucoevatromonoside (Figure S1J)	Colon	Leads to the formation of 53BP1 foci and impairs DNA damage response	[124]
	Lung	Arrests cells at the G2/M phase and down-regulates the cyclin B1 and p53 expressions	[125]
strophanthidin (Figure S1K)	Lung, breast and liver	Causes apoptosis by antagonizing the MAPK, PI3K/AKT/mTOR, and Wnt/β-catenin signaling pathways	[126]
peruvoside (Figure S1L)	Lung, breast and liver	Causes cellular apoptosis by attenuating the MAPK, PI3K/AKT/mTOR, and Wnt/β-catenin signaling pathways	[127]
	Leukemia	Up-regulates the expression of CDKN1A and triggers the activation of caspase 3,8 and PARP	[128]

**Table 2:** Anticancer effect of cardiac glycosides against different cancer cell lines.

Cancer Type	Cardiac Glycosides Used	Cell Lines	Reference
Breast	digitoxin, digoxin, ouabain, oleandrin, proscillaridin A, convallatoxin, bufalin, lanatoside C, strophanthidin, peruvoside	MCF-7, MDA-MB-231, MDA-MB-435	[79,84,88,99,106,120,126,127,131]
Cervical	digoxin, oleandrin, convallatoxin,	HeLa	[86,99,105]
Colon	oleandrin, convallatoxin, neriifolin, gitoxigenin, lanatoside C, strophanthidin,	SW480, HCT116, RKO, HT-29	[96,104,124]
Leukemia	digitoxin, UNBS1450, bufalin, peruvoside	K562, U937, HEL, MOLT-4, REH, MEG-01, HL60, TF-1, KBM5, ML-1, KG1a	[82,89,102,110,111,112,113,128,132]
Liver	digoxin, cinobufagine, proscillaridin A, lanatoside C, neriifolin, strophanthidin, peruvoside	Hep3B, HA22T, Huh-7, HepG2, Mahlavu, FOCUS	[15,45,49,123,126,127,133,134]
Lung	digitoxin, digoxin, ouabain, proscillaridin A, UNBS1450, bufalin, glucoevatromonoside, lanatoside C, strophanthidin, peruvoside	A427, A549, Calu-1, H1355, H1975, H292, HCC827, NCI-H358, SK-LU-1	[15,53,71,72,78,85,90,101,107,108,118,119,126,127,134,135]
Osteosarcoma	oleandrin, proscillaridin A, bufalin	U-2 OS, Saos-2, 143B	[98,103,121]
Pancreatic	oleandrin, bufalin	PANC-1, BXPC-3, MIA PaCa-2, CFPAC-1, SW1990	[97,114,115,116]
Prostate	anvirzel, digitoxin, digoxin, ouabain, oleandrin, proscillaridin A, bufalin, cinobufagine	PC3, C4-2, DU-145, LNCaP	[76,83,87,94,100,109]

## Data Availability

Not applicable.

## Supplementary Materials

The following are available online at https://www.mdpi.com/article/10.3390/biom11091275/s1, Figure S1: 2D structures of cardiac glycosides reported in Table 1. (A) Digitoxin, (B) Digoxin, (C) Ouabain, (D) Oleandrin, (E) Proscillaridin A, (F) Convallatoxin, (G) UNBS1450, (H) Bufalin, (I) Lanatoside C, (J) Glucoevatromonoside, (K) Strophanthidin, (L) Peruvoside.

## Abbreviations

TF	Transcription factors
FL	Fas ligand
BC	Breast cancer
Erα	Estrogen receptor α
NHR	Nuclear hormone receptor
NF-kB	Nuclear factor kappa-light-chain-enhancer of activated B cells
ZEB2	E-box binding home box 2
EMT	Epithelial-mesenchymal transition
PUMA	p53 upregulated modulator of apoptosis
SRSF3	Serine Arginine-rich splicing factor 3
HOXC10	Homeodomain—containing gene 10
HIF-1α	Hypoxia-inducible factors
FGF-2	Fibroblast growth factor
STAT-3	Signal transducer and activator of transcription
