# Supplementary material for: Emergence of Cardiac Glycosides as Potential Drugs: Current and Future Scope for Cancer Therapeutics
Source: Biomolecules. 2021 Aug 25;11(9):1275. doi: 10.3390/biom11091275 (PMC8465509; doi:10.3390/biom11091275)
Supplement: Supplementary file 1 [file biomolecules-11-01275-s001.zip › biomolecules-1352760-supplementary.pdf]

Supplementary Figure S1: 2D structures of cardiac glycosides reported in Table 1. (**A**) Digitoxin, (**B**) Digoxin, (**C**) Ouabain, (**D**) Oleandrin, (E) Proscillaridin A, (**F**) Convallatoxin, (**G**) UNBS1450, (**H)** Bufalin, (**I**) Lanatoside C, (**J**) Glucoevatromonoside, (**K**) Strophanthidin, (**L**) Peruvoside.

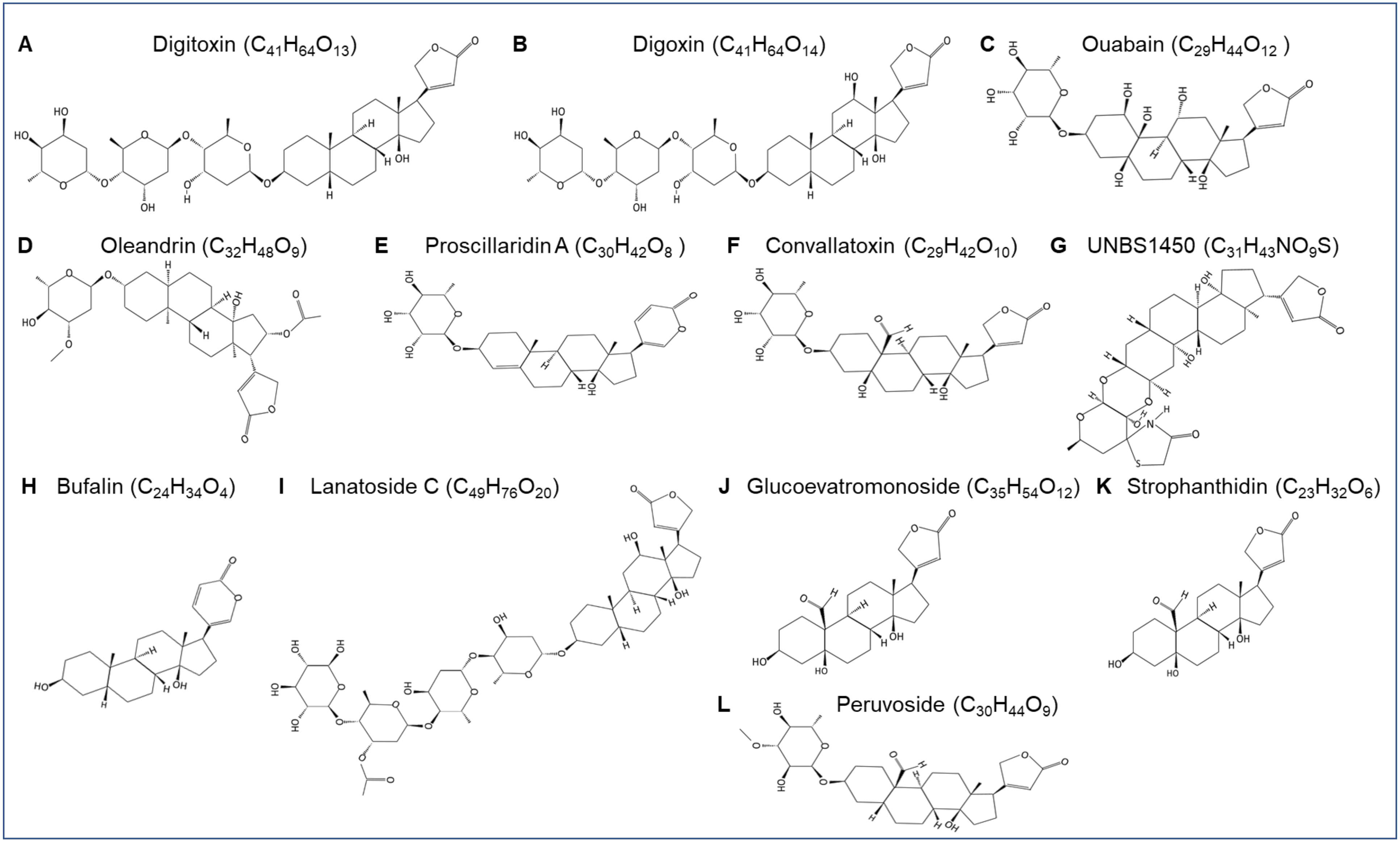

(A) digitoxin

Molecular Formula: $C_{41}H_{64}O_{13}$

IUPAC Name: 3-[(3S, 5R, 8R, 9S, 10S, 13R, 14S, 17R)-3-[(2R, 4S,5S,6R)-5-[(2S,4S,5S,6R)-5-[(2S,4S,5S,6R)-4,5-dihydroxy-6-methyloxan-2-yl]oxy-4-hydroxy-6-methyloxan-2-yl]oxy-4-hydroxy-6-methyloxan-2-yl]oxy-14-hydroxy-10,13-dimethyl-1,2,3,4,5,6,7,8,9,11,12,15,16,17-tetradecahydrocyclopenta[a]phenanthren-17-yl]-2H-furan-5-one

Isomeric SMILES: C[C@@H]1[C@H]([C@H](C[C@@H](O1)O[C@@H]2[C@H](O[C@H](C[C@@H]2O)O[C@@H]3[C@H](O[C@H](C[C@@H]3O)O[C@H]4CC[C@]5([C@@H](C4)CC[C@@H]6[C@@H]5CC[C@]7([C@@]6(CC[C@@H]7C8=CC(=O)OC8)O)C)C)C)C)O)O

InChIKey: WDJUZGPOPHTGOT-XUDUSOBPSA-N

InChI: InChI=1S/C41H64O13/c1-20-36(46)29(42)16-34(49-20)53-38-22(3)51-35(18-31(38)44)54-37-21(2)50-33(17-30(37)43)52-25-8-11-39(4)24(15-25)6-7-28-27(39)9-12-40(5)26(10-13-41(28,40)47)23-14-32(45)48-19-23/h14,20-22,24-31,33-38,42-44,46-47H,6-13,15-19H2,1-5H3/t20-,21-,22-,24-,25+,26-,27+,28-,29+,30+,31+,33+,34+,35+,36-,37-,38-,39+,40-,41+/m1/s1

(B) digoxin

Molecular Formula: $C_{41}H_{64}O_{14}$

IUPACName: 3-[(3S, 5R, 8R, 9S, 10S, 12R, 13S, 14S,17R)-3-[(2R,4S,5S,6R)-5-[(2S,4S,5S,6R)-5-[(2S,4S,5S,6R)-4,5-dihydroxy-6-methyloxan-2-yl]oxy-4-hydroxy-6-methyloxan-2-yl]oxy-4-hydroxy-6-methyloxan-2-yl]oxy-12,14-dihydroxy-10,13-dimethyl-1,2,3,4,5,6,7,8,9,11,12,15,16,17-tetradecahydrocyclopenta[a]phenanthren-17-yl]-2H-furan-5-one

IsomericSMILES: C[C@@H]1[C@H]([C@H](C[C@@H](O1)O[C@@H]2[C@H](O[C@H](C[C@@H]2O)O[C@@H]3[C@H](O[C@H](C[C@@H]3O)O[C@H]4CC[C@]5([C@@H](C4)CC[C@@H]6[C@@H]5C[C@H]([C@]7([C@@]6(CC[C@@H]7C8=CC(=O)OC8)O)C)O)C)C)C)O)O

InChIKey: LTMHDMANZUZIPE-PUGKRICDSA-N

InChI: InChI=1S/C41H64O14/c1-19-36(47)28(42)15-34(50-19)54-38-21(3)52-35(17-30(38)44)55-37-20(2)51-33(16-29(37)43)53-24-8-10-39(4)23(13-24)6-7-26-27(39)14-31(45)40(5)25(9-11-41(26,40)48)22-12-32(46)49-18-22/h12,19-21,23-31,33-38,42-45,47-48H,6-11,13-18H2,1-5H3/t19-,20-,21-,23-,24+,25-,26-,27+,28+,29+,30+,31-,33+,34+,35+,36-,37-,38-,39+,40+,41+/m1/s1

(C) ouabain

Molecular Formula: $C_{29}H_{44}O_{12}$

IUPAC Name: 3-[(1R, 3S, 5S, 8R, 9S, 10R, 11R, 13R, 14S, 17R)-1,5,11,14-tetrahydroxy-10-(hydroxymethyl)-13-methyl-3-[(2R,3R,4R,5R,6S)-3,4,5-trihydroxy-6-methyloxan-2-yl]oxy-2,3,4,6,7,8,9,11,12,15,16,17-dodecahydro-1H-cyclopenta[a]phenanthren-17-yl]-2H-furan-5-one

IsomericSMILES: C[C@H]1[C@@H]([C@H]([C@H]([C@@H](O1)O[C@H]2C[C@H]([C@@]3([C@@H]4[C@@H](CC[C@@]3(C2)O)[C@]5(CC[C@@H]([C@]5(C[C@H]4O)C)C6=CC(=O)OC6)O)CO)O)O)O)O

InChIKey: LPMXVESGRSUGHW-HBYQJFLCSA-N

InChI: InChI=1S/C29H44O12/c1-13-22 (34) 23(35) 24(36) 25(40-13) 41-15-8-19(32) 28(12-30) 21-17(3-5-27(28,37) 9-15) 29(38) 6-4-16(14-7-20(33) 39-11-14) 26(29,2) 10-18(21) 31/h7, 13,15-19,21-25,30-32,34-38H, 3-6,8-12H2, 1-2H3/t13-, 15-, 16+, 17+, 18+, 19+,21+,22-,23+,24+,25-,26+,27-,28+,29-/m0/s1

(D) oleandrin

Molecular Formula: $C_{32}H_{48}O_{9}$

IUPAC Name: [(3S, 5R, 8R, 9S, 10S, 13R, 14S, 16S, 17R)-14-hydroxy-3-[(2R,4S,5S,6S)-5-hydroxy-4-methoxy-6-methyloxan-2-yl]oxy-10,13-dimethyl-17-(5-oxo-2H-furan-3-yl)-1,2,3,4,5,6,7,8,9,11,12,15,16,17-tetradecahydrocyclopenta[a]phenanthren-16-yl] acetate

IsomericSMILES: C[C@H]1[C@@H]([C@H](C[C@@H](O1)O[C@H]2CC[C@]3([C@@H](C2)CC[C@@H]4[C@@H]3CC[C@]5([C@@]4(C[C@@H]([C@@H]5C6=CC(=O)OC6)OC(=O)C)O)C)C)OC)O

InChIKey: JLPDBLFIVFSOCC-XYXFTTADSA-N

InChI: InChI=1S/C32H48O9/c1-17-29(35)24(37-5)14-27(39-17)41-21-8-10-30(3)20(13-21)6-7-23-22(30)9-11-31(4)28(19-12-26(34)38-16-19)25(40-18(2)33)15-32(23,31)36/h12,17,20-25,27-29,35-36H,6-11,13-16H2,1-5H3/t17-,20+,21-,22-,23+,24-,25-,27-,28-,29-,30-,31+,32-/m0/s1

(E) proscillaridin A

Molecular Formula: $C_{30}H_{42}O_{8}$

IUPAC Name: 5-[(3S, 8R, 9S, 10R, 13R, 14S, 17R)-14-hydroxy-10, 13-dimethyl-3-[(2R,3R,4R,5R,6S)-3,4,5-trihydroxy-6-methyloxan-2-yl]oxy-1,2,3,6,7,8,9,11,12,15,16,17-dodecahydrocyclopenta[a]phenanthren-17-yl]pyran-2-one

IsomericSMILES: C[C@H]1[C@@H]([C@H]([C@H]([C@@H](O1)O[C@H]2CC[C@@]3([C@H]4CC[C@@]5([C@H](CC[C@@]5([C@@H]4CCC3=C2)O)C6=COC(=O)C=C6)C)C)O)O)O

InChIKey: MYEJFUXQJGHEQK-ALRJYLEOSA-N

InChI: InChI=1S/C30H42O8/c1-16-24(32)25(33)26(34)27(37-16)38-19-8-11-28(2)18(14-19)5-6-22-21(28)9-12-29(3)20(10-13-30(22,29)35)17-4-7-23(31)36-15-17/h4,7,14-16,19-22,24-27,32-35H,5-6,8-13H2,1-3H3/t16-,19-,20+,21-,22+,24-,25+,26+,27-,28-,29+,30-/m0/s1

(F) Convallatoxin

Molecular Formula: $C_{29}H_{42}O_{10}$

IUPAC Name: (3S, 5S, 8R, 9S, 10S, 13R, 14S, 17R)-5,14-dihydroxy-13-methyl-17-(5-oxo-2H-furan-3-yl)-3-[(2R,3R,4R,5R,6S)-3,4,5-trihydroxy-6-methyloxan-2-yl]oxy-2,3,4,6,7,8,9,11,12,15,16,17-dodecahydro-1H-cyclopenta[a]phenanthrene-10-carbaldehyde

IsomericSMILES: C[C@H]1[C@@H]([C@H]([C@H]([C@@H](O1)O[C@H]2CC[C@@]3([C@H]4CC[C@@]5([C@H](CC[C@@]5([C@@H]4CC[C@@]3(C2)O)O)C6=CC(=O)OC6)C)C=O)O)O)O

InChIKey: HULMNSIAKWANQO-JQKSAQOKSA-N

InChI: InChI=1S/C29H42O10/c1-15-22(32)23(33)24(34)25(38-15)39-17-3-8-27(14-30)19-4-7-26(2)18(16-11-21(31)37-13-16)6-10-29(26,36)20(19)5-9-28(27,35)12-17/h11,14-15,17-20,22-25,32-36H,3-10,12-13H2,1-2H3/t15-,17-,18+,19-,20+,22-,23+,24+,25-,26+,27-,28-,29-/m0/s1

(G) UNBS1450

Molecular Formula: $C_{31}H_{43}NO_9S$

IUPAC Name: (1'S, 3'R, 5'S, 7'R, 10'R, 12'R, 14'R, 15'S, 18'R, 19'R, 22'S, 23'R)-10',22'-dihydroxy-14'-(hydroxymethyl)-7',18'-dimethyl-19'-(5-oxo-2H-furan-3-yl)spiro[1,3-thiazolidine-2,9'-4,6,11-trioxahexacyclo[12.11.0.03,12.05,10.015,23.018,22]pentacosane]-4-one

IsomericSMILES: C[C@@H]1CC2([C@]3([C@@H](O1)O[C@@H]4C[C@@H]5CC[C@@H]6[C@@H]([C@]5(C[C@H]4O3)CO)CC[C@]7([C@@]6(CC[C@@H]7C8=CC(=O)OC8)O)C)O)NC(=O)CS2

InChIKey: GRINEMOQBRCRRY-PNHWRJPOSA-N

InChI: InChI=1S/C31H43NO9S/c1-16-11-30(32-24(34)14-42-30)31(37)26(39-16)40-22-10-18-3-4-21-20(28(18,15-33)12-23(22)41-31)5-7-27(2)19(6-8-29(21,27)36)17-9-25(35)38-13-17/h9,16,18-23,26,33,36-37H,3-8,10-15H2,1-2H3,(H,32,34)/t16-,18+,19-,20+,21-,22-,23-,26+,27-,28-,29+,30?,31-/m1/s1

(H) bufalin

Molecular Formula: $C_{24}H_{34}O_4$

IUPAC Name: 5-[(3S, 5R, 8R, 9S, 10S, 13R, 14S, 17R)-3,14-dihydroxy-10, 13-dimethyl-1, 2,3,4,5,6,7,8,9,11,12,15,16,17-tetradecahydrocyclopenta [a] phenanthren-17-yl] pyran-2-one

Isomeric SMILES: C[C@]12CC[C@@H](C[C@H]1CC[C@@H]3[C@@H]2CC[C@]4([C@@]3(CC[C@@H]4C5=COC(=O)C=C5)O)C)O

InChIKey: QEEBRPGZBVVINN-BMPKRDENSA-N

InChI: InChI=1S/C24H34O4/c1-22-10-7-17(25)13-16(22)4-5-20-19(22)8-11-23(2)18(9-12-24(20,23)27)15-3-6-21(26)28-14-15/h3,6,14,16-20,25,27H,4-5,7-13H2,1-2H3/t16-,17+,18-,19+,20-,22+,23-,24+/m1/s1

(I) **lanatoside C**

Molecular Formula: $C_{49}H_{76}O_{20}$

IUPAC Name: [(2R, 3R, 4S,6S)-6-[(2R,3S,4S,6S)-6-[(2R,3S,4S,6R)-6-[[(3S,5R,8R,9S,10S,12R,13S,14S,17R)-12,14-dihydroxy-10,13-dimethyl-17-(5-oxo-2H-furan-3-yl)-1,2,3,4,5,6,7,8,9,11,12,15,16,17-tetradecahydrocyclopenta[a]phenanthren-3-yl]oxy]-4-hydroxy-2-methyloxan-3-yl]oxy-4-hydroxy-2-methyloxan-3-yl]oxy-2-methyl-3-[(2S,3R,4S,5S,6R)-3,4,5-trihydroxy-6-(hydroxymethyl)oxan-2-yl]oxyoxan-4-yl] acetate

IsomericSMILES: C[C@@H]1[C@H]([C@H](C[C@@H](O1)O[C@H]2CC[C@]3([C@@H](C2)CC[C@@H]4[C@@H]3C[C@H]([C@]5([C@@]4(CC[C@@H]5C6=CC(=O)OC6)O)C)O)C)O)O[C@H]7C[C@@H]([C@@H]([C@H](O7)C)O[C@H]8C[C@@H]([C@@H]([C@H](O8)C)O[C@H]9[C@@H]([C@H]([C@@H]([C@H](O9)CO)O)O)O)OC(=O)C)O

InChIKey: JAYAGJDXJIDEKI-PTGWOZRBSA-N

InChI: InChI=1S/C49H76O20/c1-21-43(67-38-17-32(53)44(22(2)62-38)68-39-18-33(64-24(4)51)45(23(3)63-39)69-46-42(58)41(57)40(56)34(19-50)66-46)31(52)16-37(61-21)65-27-9-11-47(5)26(14-27)7-8-29-30(47)15-35(54)48(6)28(10-12-49(29,48)59)25-13-36(55)60-20-25/h13,21-23,26-35,37-46,50,52-54,56-59H,7-12,14-20H2,1-6H3/t21-,22-,23-,26-,27+,28-,29-,30+,31+,32+,33+,34-,35-,37+,38+,39+,40-,41+,42-,43-,44-,45-,46+,47+,48+,49+/m1/s1

(J) glucoevatromonoside

Molecular Formula: $C_{35}H_{54}O_{12}$

IUPAC Name: 3-[(3S, 5R, 8R, 9S, 10S, 13R, 14S, 17R)-14-hydroxy-3-[(2R,4S,5S,6R)-4-hydroxy-6-methyl-5-[(2S,3R,4S,5S,6R)-3,4,5-trihydroxy-6-(hydroxymethyl)oxan-2-yl]oxyoxan-2-yl]oxy-10,13-dimethyl-1,2,3,4,5,6,7,8,9,11,12,15,16,17-tetradecahydrocyclopenta[a]phenanthren-17-yl]-2H-furan-5-one

IsomericSMILES: C[C@@H]1[C@H]([C@H](C[C@@H](O1)O[C@H]2CC[C@]3([C@@H](C2)CC[C@@H]4[C@@H]3CC[C@]5([C@@]4(CC[C@@H]5C6=CC(=O)OC6)O)C)C)O)O[C@H]7[C@@H]([C@H]([C@@H]([C@H](O7)CO)O)O)O

InChIKey: BKLVUVLBSZAKIF-DQEYRRRMSA-N

InChI: InChI=1S/C35H54O12/c1-17-31(47-32-30(41)29(40)28(39)25(15-36)46-32)24(37)14-27(44-17)45-20-6-9-33(2)19(13-20)4-5-23-22(33)7-10-34(3)21(8-11-35(23,34)42)18-12-26(38)43-16-18/h12,17,19-25,27-32,36-37,39-42H,4-11,13-16H2,1-3H3/t17-,19-,20+,21-,22+,23-,24+,25-,27+,28-,29+,30-,31-,32+,33+,34-,35+/m1/s1

(K) strophanthidin

Molecular Formula: $C_{23}H_{32}O_6$

IUPAC Name: (3S, 5S, 8R, 9S, 10S, 13R, 14S, 17R)-3,5,14-trihydroxy-13-methyl-17-(5-oxo-2H-furan-3-yl)-2,3,4,6,7,8,9,11,12,15,16,17-dodecahydro-1H-cyclopenta[a]phenanthrene-10-carbaldehyde

IsomericSMILES: C[C@]12CC[C@H]3[C@H]([C@]1(CC[C@@H]2C4=CC(=O)OC4)O)CC[C@]5([C@@]3(CC[C@@H](C5)O)C=O)O

InChIKey: ODJLBQGVINUMMR-HZXDTFASSA-N

InChI: InChI=1S/C23H32O6/c1-20-6-3-17-18(4-8-22(27)11-15(25)2-7-21(17,22)13-24)23(20,28)9-5-16(20)14-10-19(26)29-12-14/h10,13,15-18,25,27-28H,2-9,11-12H2,1H3/t15-,16+,17-,18+,20+,21-,22-,23-/m0/s1

(L) peruvoside

Molecular Formula: $C_{30}H_{44}O_9$

IUPAC Name: (3S, 5R, 8R, 9S, 10R, 13R, 14S, 17R)-3-[(2R, 3S, 4R, 5S, 6S)-3,5-dihydroxy-4-methoxy-6-methyloxan-2-yl] oxy-14-hydroxy-13-methyl-17-(5-oxo-2H-furan-3-yl)-1,2,3,4,5,6,7,8,9,11,12,15,16,17-tetradecahydrocyclopenta [a]phenanthrene-10-carbaldehyde

IsomericSMILES: C[C@H]1[C@@H]([C@H]([C@@H]([C@@H](O1)O[C@H]2CC[C@]3([C@@H](C2)CC[C@@H]4[C@@H]3CC[C@]5([C@@]4(CC[C@@H]5C6=CC(=O)OC6)O)C)C=O)O)OC)O

InChIKey: PMTSPAGBAFCORP-HBUONDEYSA-N

InChI: InChI=1S/C30H44O9/c1-16-24(33)26(36-3)25(34)27(38-16)39-19-6-10-29(15-31)18(13-19)4-5-22-21(29)7-9-28(2)20(8-11-30(22,28)35)17-12-23(32)37-14-17/h12,15-16,18-22,24-27,33-35H,4-11,13-14H2,1-3H3/t16-,18+,19-,20+,21-,22+,24-,25-,26+,27-,28+,29+,30-/m0/s1
